# Impact of Submarine Groundwater Discharge on Marine Water Quality and Reef Biota of Maui

**DOI:** 10.1371/journal.pone.0165825

**Published:** 2016-11-03

**Authors:** Daniel W. Amato, James M. Bishop, Craig R. Glenn, Henrietta Dulai, Celia M. Smith

**Affiliations:** 1 Department of Botany, University of Hawaiʻi at Mānoa, Honolulu, Hawaiʻi, United States of America; 2 Department of Geology and Geophysics, University of Hawaiʻi at Mānoa, Honolulu, Hawaiʻi, United States of America; 3 US Geological Survey, Menlo Park, California, United States of America; Northwest Fisheries Science Center, UNITED STATES

## Abstract

Generally unseen and infrequently measured, submarine groundwater discharge (SGD) can transport potentially large loads of nutrients and other land-based contaminants to coastal ecosystems. To examine this linkage we employed algal bioassays, benthic community analysis, and geochemical methods to examine water quality and community parameters of nearshore reefs adjacent to a variety of potential, land-based nutrient sources on Maui. Three common reef algae, *Acanthophora spicifera*, *Hypnea musciformis*, and *Ulva* spp. were collected and/or deployed at six locations with SGD. Algal tissue nitrogen (N) parameters (δ^15^N, N %, and C:N) were compared with nutrient and δ^15^N-nitrate values of coastal groundwater and nearshore surface water at all locations. Benthic community composition was estimated for ten 10-m transects per location. Reefs adjacent to sugarcane farms had the greatest abundance of macroalgae, low species diversity, and the highest concentrations of N in algal tissues, coastal groundwater, and marine surface waters compared to locations with low anthropogenic impact. Based on δ^15^N values of algal tissues, we estimate ca. 0.31 km^2^ of Kahului Bay is impacted by effluent injected underground at the Kahului Wastewater Reclamation Facility (WRF); this region is barren of corals and almost entirely dominated by colonial zoanthids. Significant correlations among parameters of algal tissue N with adjacent surface and coastal groundwater N indicate that these bioassays provided a useful measure of nutrient source and loading. A conceptual model that uses *Ulva* spp. tissue δ^15^N and N % to identify potential N source(s) and relative N loading is proposed for Hawaiʻi. These results indicate that SGD can be a significant transport pathway for land-based nutrients with important biogeochemical and ecological implications in tropical, oceanic islands.

## Introduction

Connections between land use, coastal water quality, and marine ecosystem health are often difficult to identify because multiple pathways may influence nutrient loading to coastal water bodies [1]. While the effects of terrestrial runoff on marine ecosystems have been characterized widely [2, 3], relatively little is known about the interaction between submarine groundwater discharge (SGD) and nearshore marine communities. It is clear that SGD is a significant source of N to the sea regardless of the level of human impact on the adjacent land [4–7]; this is particularly true for tropical, oligotrophic regions [8–11]. In areas where the nutrient concentration of coastal groundwater (CGW) has been increased by anthropogenic activities, nutrient loading to coastal waters via SGD has been associated with macroalgal blooms and shifts in the composition of biological communities [12–15], harmful algal (phytoplankton) blooms [16–18], and eutrophication [5, 19] in coastal ecosystems worldwide.

On Maui, coral reef health has suffered from the synergistic effects of nutrient pollution, overfishing, and invasive species [20–22]. Persistent blooms of opportunistic macroalgae, specifically *Hypnea musciformis*, *Ulva lactuca*, and *Cladophora sericea*, have occurred in coastal areas proximal to wastewater treatment facilities that use injection wells for effluent disposal and/or proximal to regions with large-scale agriculture [23–28]. The most obvious and direct deleterious impacts of macroalgae on corals are observed when algae overgrow and physically disturb corals while competing for light, nutrients, and space [29–33]. In addition, both nutrient loading and algae may indirectly affect corals via other biochemical pathways [34]. The presence of algae, even in absence of direct physical contact, can increase coral disease and mortality through the release of dissolved compounds that enhance microbial activity on coral tissues [35].

Using a combination of algal bioassays, geochemical modeling (including dye-tracers), and water sampling, recent studies on Maui have shown a clear link between municipal wastewater injection wells, SGD, and nearshore reefs in Kihei and Lahaina [8–10, 36, 37]. As a bioassay, the use of algal tissue δ^15^N values (and other benthic biota) is well established and particularly useful in monitoring the extent of wastewater pollution in a variety of coastal, but especially tropical, oligotrophic environments [3, 8, 9, 37–46]. Sewage effluent and other denitrified sources are generally enriched in ^15^N relative to ^14^N because of the preferential use of the lighter N isotope (^14^N) by bacteria [47]. Published δ^15^N values of wastewater-derived N range from +7 ‰ to +93 ‰ for nitrate dissolved in water [10, 36, 37, 48], and from +4 ‰ to +50 ‰ in marine macroalgal tissue [8, 10, 36, 42–44]. Natural and synthetic fertilizer-based N sources generally have low δ^15^N values (0 ‰ to +4 ‰ and -4 ‰ to +4 ‰, respectively) [48, 49], allowing for identification of wastewater sources to be relatively straightforward. In contrast, unequivocal identification of some fertilizer-derived N may be confounded by inputs from other sources with similar, low δ^15^N values [48]. While δ^15^N values do not imply N amount, amount of N in algal tissues (N %) and the tissue C:N ratio have been used as a relative indicator of biologically available N in water and N limitation in algae, respectively [44, 50, 51]. The parameter algal tissue N (%) provides a sound measure of available N at a location because N is integrated over the period of incubation and that tissues can incorporate nutrients from pulses associated with tidal processes (such as SGD) or runoff events that may not be detected with conventional water sampling [52].

The purpose of this study was to test for links among land use, N content of CGW, water quality of marine surface waters, and reef biota at locations where SGD input and associated nutrient sources were characterized via a concurrent study [53]. Using common marine algae as bioindicators of available nitrogen in coastal settings, we hypothesized that algal tissue N (δ^15^N and N %) would be related to the dominant N source and amount of biologically available N in CGW and marine surface waters of Maui. Our results are discussed in the context of SGD as a significant pathway for anthropogenic nutrient loading and the potential impacts of SGD nutrient loading on reef ecosystems.

## Methods

### Study locations

**Fig 1:**
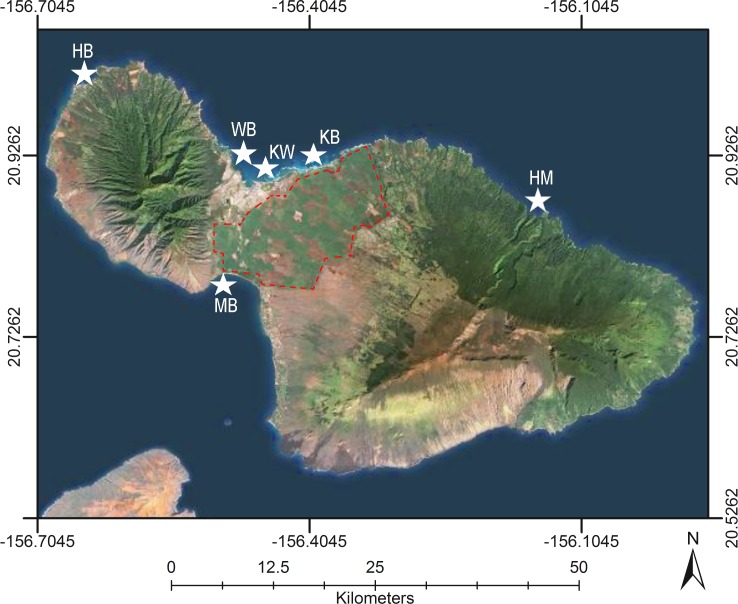
Study locations on Maui. Study locations Kahului (KW), Kūʻau (KB), Māʻalaea (MB), Honomanū (HM), Honolua (HB), and Waiehu (WB) Bays are shown as stars. The red, dotted line indicates the boundary of current sugarcane fields. Satellite imagery was used with permission from Esri (DigitalGlobe, GeoEye, i-cubed, Earthstar Geographics, CNES/Airbus DS, USDA, USGS, AEX, Getmapping, Aerogrid, IGN, IGP, swisstopo, and the GIS User Community; All rights reserved).

Six locations were chosen for this study to represent various land use and potential sources of nutrients on Maui (Fig 1). All locations showed precursory evidence of SGD as indicated by lowered nearshore salinity unassociated with surface runoff. Honomanū Bay (NE Maui, Latitude 20.86219, Longitude -156.165667; HM on Fig 1), initially judged to be the least human-affected location because of its remote location, has no known anthropogenic land-based sources of contamination or history of algal blooms. Tropical forest dominates the watershed of this bay and a groundwater-fed stream discharges to the NW portion of the bay. Honolua Bay (NW Maui, Latitude 21.013829, Longitude -156.639656; HB on Fig 1) is a designated marine life conservation district (MLCD) that is surrounded by a moderately impacted watershed with potential nutrient sources that include golf courses and low-density onsite sewage disposal systems OSDS [54]. Pineapple fields dominated nearby lands above Honolua Bay for over nine decades, but have been fallow since 2006. An intermittent stream at the center of Honolua Bay is a source of sediment and organic debris from the watershed during large rain events [21]. Previous studies indicate that Honolua Bay has a relatively intact marine grazer community with fish biomass that is comparable to other MLCD sites [55]. Both Honomanū and Honolua Bays are relatively small, narrow, and have deep, central channels compared to the other study locations. Steep cliffs flank both sides of a small beach at the center of these bays.

Waiehu, Kahului, Kūʻau, and Māʻalaea Bays represent study locations in which moderate to high levels of nutrient flux were likely as judged by the land use features: presence of OSDS, municipal wastewater injection facility, and/or large-scale agriculture. All four locations had abundant invasive algal species (*Ulva lactuca*, *Hypnea musciformis*, and/or *Acanthophora spicifera*) in the intertidal to subtidal zone. Waiehu Bay (N Maui, Latitude 20.914023, Longitude -156.489007; WB in Fig 1) is a relatively shallow, moderately sized bay with a sandy beach fronting a wetland at its center. A small stream that drains macadamia tree plantations upslope discharges at the northern tip of the bay. An additional potential source of N to Waiehu Bay was an adjacent residential area with a greater density of OSDS compared to other study locations [54]. Similar in size and depth to Waiehu Bay, Kūʻau Bay (N Maui, Latitude 20.926169, Longitude -156.373436; KB in Fig 1) is surrounded by extensive sugarcane agriculture within 100 m of the shoreline. Although OSDS units occur at a relatively low density in this coastal area, high-density OSDS upslope was identified as an additional source of nutrients to Kūʻau Bay [54]. The shoreline itself is comprised of basalt boulders and a sand beach at the NW corner.

The Kahului Bay (N Maui, Latitude 20.898709, Longitude -156.455962; KW in Fig 1) study location was adjacent to the Kahului WRF. This facility uses eight wastewater injection wells located within 50 m of the shoreline to dispose 16,800 m^3^ d^-1^ (4.4 million gal d^-1^) treated sewage into CGW [8]. Although there is no agriculture adjacent to the coast in this area, groundwater upgradient from this location may also be impacted by sugarcane production and low density of OSDS [54]. Kahului Bay is a large water body with a shallow nearshore reef and an extensive sand beach relative to Honomanū and Honolua Bays. Māʻalaea Bay (S Maui, Latitude 20.792548, Longitude -156.508104; MB in Fig 1) lies directly south of extensive sugarcane agriculture and has shallow wastewater injection wells that service the numerous condominiums located near the coastline [8, 27]. Direct discharge of water (from Maui Ocean Center and Maui Electric Company) with low N concentrations [8] and low-density OSDS [54] are potential sources of nutrients to nearshore reefs in Māʻalaea Bay. Similar to Kahului Bay, Māʻalaea Bay is a large water body with a relatively shallow nearshore reef. At the NE corner of the bay, salt marshes at Keālia Pond National Wildlife Refuge provide a buffer between surgarcane fields and a sandy beach. A concurrent study, Bishop et al. [53] provided estimates of SGD-derived nutrient fluxes, groundwater flow paths, and land use assessments for all these study locations.

No specific permissions were required for these research activities at the study locations. All sampling took place within public areas in collaboration with the Division of Aquatic Resources, State of Hawaiʻi; In Hawaiʻi, the public has the right of access along shorelines situated below the “upper reaches of the wash of the waves.” No algae were removed from Honolua Bay MLCD and a permit to use state submerged lands was not required for activities (cage deployments) less than two weeks.

### Algal bioassays

Tissues of common marine algae were used to assess the availability of inorganic N in coastal waters with two approaches: 1) shore-collected samples via collection of *in situ* algae from intertidal and nearshore subtidal zones and 2) deployed plants that were pretreated and deployed for a short-term in anchored cages following Dailer et al. [8, 9]. Individuals of *Ulva lactuca* were collected from the intertidal zone at Ke‘ahamoe Bay, O‘ahu. Prior to deployment, *U*. *lactuca* tissues were pretreated in low-nutrient artificial seawater for one week (Instant Ocean® Sea Salt and distilled water to a salinity of 35 ‰) to draw down tissue N. Samples were exposed to filtered natural sunlight (translucent glass) at a maximum of ~ 700 μM photons m^-2^ s^-1^ photosynthetically active radiation (4π Li-Cor^®^ quantum sensor, Model LI-193SA, Li-Cor, NE, USA) and aeration. Reagent grade sodium nitrate and sodium phosphate were added every two days in addition to distilled water to maintain water nutrient and salinity levels typical of oligotrophic coastal waters: final concentrations were 0.2 μM NO_3_^¯^, 0.05 μM PO_4_^3¯^ at 35 ‰ salinity [56]. To quantify initial tissue chemistry following this pre-treatment phase, nine samples (three per deployment period) were prepared for tissue N and C analyses prior to bioassay deployment: tissues were triple rinsed in distilled water, holdfast tissues and fouling organisms were removed, vegetative tissues were blotted dry with paper towel, placed in an aluminum foil tray, and dried at 71°C in a conventional oven for at least one week. Algal samples were then transported back to the University of Hawaiʻi at Mānoa (UHM), stored at 60°C until a constant mass was achieved, ground to powder, and placed in individual glass vials that were stored in a desiccant until analysis at the UHM Biogeochemical Stable Isotope Facility (BSIF) for determinations of tissue δ^15^N (‰), N %, and C:N (ratio of mass) using a Costech ECS 4010 Elemental Combustion System (Costech Analytical Technologies, CA, USA) interfaced with a ThermoFinnigan DeltaXP (Thermo Fisher Scientific Inc., MA, USA). Isotopic compositions of N in samples were normalized to reference materials NIST 3, USGS-32, USGS-34, and USGS-35 relative to AIR. Ratios of ^15^N:^14^N are expressed as δ^15^N in per mil (‰) and were calculated using Eq 1 [57].

$$\delta {}^{15}\text{N}\;\left(‰\right)=\left\{\left(\frac{R_{sample}}{R_{standard}}\right)-1\right\}\;x\;10^{3}\;\text{where}\;R=\frac{{}^{15}N}{{}^{14}N}$$ (1)

For the algal deployment bioassay, three individuals of *Ulva lactuca* (with intact holdfasts and no signs of reproductive or necrotic tissue) were placed in a cylindrical cage (8 cm x 20 cm) that was constructed of 8 mm diameter plastic mesh and polyester fabric. This allowed water flow and excluded macroherbivores. Cages were suspended 0.25 m below the sea surface on a single line tethered to a cinder block anchor and small float at each site for 5 to 6 days. Depending on the area of the study location, 8 to 16 cages were deployed during three deployment periods of two locations per period.

For the shore-collected algal bioassays, three individuals of *Ulva lactuca*, *Hypnea musciformis*, and *Acanthophora spicifera* were collected, when present, from the intertidal zone or shallow nearshore reef (< 3 m depth). Algal deployment and shore-collections were completed during July and August of 2012 at Māʻalaea and Honolua Bays, and during July of 2013 at Kūʻau, Honomanū, Kahului, and Waiehu Bays. After sample retrieval of both deployed and shore-collected tissues, all plants were immediately prepared for tissue analysis as described above. For each algal collection/deployment site, one individual algal sample was submitted to BSIF with the exception of Māʻalaea Bay shore-collected samples, where three samples per collection site were submitted to BSIF to measure variability among sample values at a single site. Analytical error was calculated as the average error between duplicates (the absolute value of the difference between duplicate samples expressed as a percentage of the mean of duplicate sample values) for algal tissue N parameters using 23 duplicate pairs.

Species of *Ulva* have variable morphologies and uncertain identities in field collections [58]. 13 samples were collected for deployment between 2012 and 2013 and submitted to the Algal Biodiversity Lab at UHM for post-experimental molecular identification. Comparisons of sample primary sequence data and ITS1 secondary structure with the results of O’Kelly et al. [58] identified three operational taxonomic units with sequence matches to species *Ulva lactuca* and *Ulva ohnoi*. Hereafter, samples with *Ulva lactuca*-type morphology are referred to as *Ulva*.

### Water samples

Marine surface water samples (0.25 m depth) were collected during the 2012 and 2013 algal cage deployments at sites adjacent to all cages, in addition to select nearshore sites, at low tide. A piezometer and peristaltic pump were used to collect CGW above the swash zone in all study locations during algal deployments and at select sites in late March to early April of 2014. In this work, we define CGW as water (fresh to saline) obtained from shallow beach pore water or distinct coastal springs. CGW is assumed to be representative of the composition of the SGD endmember prior to release in the ocean. A sample of treated Kahului WRF effluent was also obtained. The salinity of all samples was measured using a YSI multiparameter sonde (Yellow Springs Instruments, model V24 6600 with conductivity/temperature sensor Model 6560, OH, USA). Samples were initially collected in acid washed 500-ml bottles and stored on ice for up to 12 h.

All water samples were analyzed for total dissolved nitrogen (TDN), total dissolved phosphorus (TDP), and dissolved inorganic nutrients (SiO_4_^4-^, NO_3_^-^, NO_2_^-^, NH_4_^+^, and PO_4_^3-^) at the SOEST Laboratory for Analytical Biogeochemistry (S-LAB) at UHM using a Seal Analytical AA3 Nutrient Autoanalyzer. In order to calculate average nutrient concentrations at a given location, concentrations below the level of detection were designated as zero. The isotopic composition of N in dissolved NO_3_^-^ was measured at BSIF following the denitrifier method [59] with a Finnigan MAT252 coupled to a GasBench II (Thermo Fisher Scientific Inc., MA, USA) for samples with NO_3_^-^ ≥ 1 μM and expressed as δ^15^N-NO_3_^-^ following Eq 1. For samples that had a concentration of NO_2_^-^ greater than 1% of the nitrate concentration, NO_2_^-^ was removed using sulfamic acid during sample preparation [60]. In addition to the international N standards listed above, an in-house NaNO_3_ standard was used to characterize the δ^15^N-NO_3_^-^ of water samples. Only water samples collected during *Ulva* deployments (2012 to 2013) were included in the δ^15^N-NO_3_^-^ analysis, with the exception of water samples collected during 2014 (δ^15^N-NO_3_^-^ values for Māʻalaea and Honolua Bays were not available for 2012–2013) under similar SGD and ocean conditions. A set of duplicate water samples was submitted for δ^15^N-NO_3_^-^ (n = 15 duplicate pairs) and nutrient analysis (n = 22 duplicate pairs) to estimate analytical error as described above.

### Coastal benthic community analyses

Analysis of the marine benthic community followed the state-wide Coral Reef Assessment and Monitoring Program’s (CRAMP) rapid assessment protocol [61, 62] during March and April of 2014. Two adjacent nearshore areas were selected at each field location, within a 1 to 3 m depth for benthic analysis. Within each area, a 4 m x 100 m transect grid (50 potential, 10-m shore-parallel transects) was generated using ArcMap 10.0 (ESRI, CA, USA). Five transects were randomly chosen from the grid for benthic analysis using a random number generator. A Nikon AW110 camera attached to a PVC photoquadrat frame (18.2 cm × 27.0 cm) was used to take one photograph every meter per transect for a total of 100 images per study location.

Each image represented 458.8 cm^2^ of benthic surface after being cropped to a size of 3,148 x 2,010 pixels to remove the PVC frame. PhotoGrid software [63] was used to analyze each image using a point-intercept method with 25 random points per image. Benthic organisms or substrates were identified to the best taxon level possible and grouped into categories: coral, macroalgae, turf algae, crustose coralline algae, invertebrates, and abiotic substrate. The proportion of points in each category (p_i_) in reference to total points per transect (250 points) and per location (2500 points) was calculated. In addition to species richness (sum of unique species), Shannon’s diversity (H’) index [64] and Simpson’s dominance (λ) index [65] were calculated with Eqs 2 and 3, respectively.

$$\mathrm{Shannon’s Diversity}\;\left(\mathrm{H’}\right)=-\sum_{i=1}^{R}{\text{p}}_{i}\times {\mathrm{ln p}}_{i}$$ (2)

$$Simpson’s\;Dominance\;(\lambda )=\sum_{i=1}^{R}p_{i}^{2}$$ (3)

### Geospatial and statistical analyses

GPS coordinates (Datum = WGS 1984) of algal and water sample sites were imported to ArcMap with associated sample data to produce maps of each study location. The distance (m) from *Ulva* deployment sites to the shoreline, or to the Kahului WRF, was calculated in ArcGIS using shoreline data provided by the State of Hawaiʻi [66]. Water and algal tissue parameters were spatially joined with those of their nearest neighbor within a 100 m radius using ArcMap. Similar to other studies [8, 46, 67], we present mean algal tissue δ^15^N values for shoreline collection sites with more than one species (Kūʻau and Kahului Bays), because minimal variation in tissue δ^15^N was found among species at identical sites. ArcMap was used to interpolate algal tissue δ^15^N values from both deployed and shore-collected samples at Kahului Bay using an ordinary kriging method with a spherical semivariogram model and a variable search radius. The estimated extent of the Kahului WRF wastewater injection plume, as shown in Burnham et al. [68], was overlain on an aerial image using the shoreline as a reference. To estimate the area affected by wastewater-derived N, ArcMap was used to calculate the area of polygons where interpolated algal tissue δ^15^N values were ≥ 8 ‰. This was a more conservative (higher) value than previously published algal tissue and dissolved nitrate δ^15^N values that have been used to indicate sewage-derived N as discussed above.

SigmaPlot 11 (Systat Software Inc., CA, USA) was used to perform all statistical tests. Nonparametric tests such as Kruskal-Wallis ANOVA (identified by the H-statistic), Dunn’s pairwise comparisons, and Spearman’s correlations (identified by correlation coefficient r_s_) were performed if the assumptions of normality or homoscedasticity were violated. One-way ANOVA (identified by the F-statistic), Tukey’s pairwise comparisons, and ordinary least square regressions were used to compare parameters if test assumptions were not violated.

## Results

### Relationships between water and algal tissue nutrients

Following the hydrological characterization by Bishop et al. [53], SGD inputs were present at all locations as evidenced by reduced salinity and elevated radon in nearshore waters relative to offshore sites during the period of this study. Further, gradients were observed in marine surface water at all locations with lower salinity and higher nutrient concentrations at nearshore sites compared to offshore sites. In general, nutrients in surface water sampled at algal deployment sites had strong inverse correlations with distance from shore and increasing salinity (S1–S7 Tables).

Concentrations of dissolved inorganic nitrogen (DIN) in marine surface water and CGW were significantly correlated with N % values and C:N ratios of algal tissues from both shore-collected and deployed samples (Table 1). Similar correlations were found for δ^15^N-NO_3_^-^ values of marine surface water and CGW with tissue δ^15^N values of both shore-collected and deployed algae (Table 2). Mean analytical error of duplicate algal samples was 5.2% (0.17 ‰ average difference between duplicates) for tissue δ^15^N values, 2.5% for tissue N, and 1.0% for tissue C:N. Mean analytical error for nutrients in water samples nutrients was ± 1.8% (SiO_4_^4-^), 12.0% (NO_3_^-^), 18.0% (NO_2_^-^), 67.7% (NH_4_^+^), 9.2% (PO_4_^3-^), 13.0% (TDN), and 18.8% (TDP). Mean analytical error for δ^15^N-NO_3_^-^ values was ± 7.4% with an average difference of 0.15 ‰ between duplicates.

**Table 1:** Spearman’s correlation results for algal tissue N % and C:N vs. water DIN (μM).

		DeployedN %	Deployed C:N	Shore-collected N %	Shore-collected C:N
**Surface DIN**	r_s_	0.596	-0.661	0.8	-0.818
	p	2.76 x10^-7^	2 x10^-7^	2 x10^-7^	2 x10^-7^
	n	63	63	36	36
**CGW DIN**	r_s_	0.75	-0.77	0.726	-0.839
	p	2 x10^-7^	2 x10^-7^	2 x10^-7^	2 x10^-7^
	n	29	29	35	35

Dissolved inorganic nitrogen (DIN) concentrations of marine surface water (Surface DIN) and coastal groundwater (CGW DIN) were correlated with both deployed *Ulva* and shore-collected tissue values of N % and C:N. Spearman’s correlation coefficient is shown as r_s_, the p-value is shown as p, and n represents sample size.

**Table 2:** Spearman’s correlation results for algal tissue δ^15^N values vs. water δ^15^N-NO_3_^-^.

		Deployed δ^15^N	Shore-collected δ^15^N
**Surface δ**^**15**^**N**	r_s_	0.74	0.529
	p	1.09 x10^-4^	1.15 x10^-2^
	n	19	22
**CGW δ**^**15**^**N**	r_s_	0.402	0.632
	p	3.07 x10^-2^	4.68 x10^-5^
	n	29	35

δ^15^N-NO_3_^-^ (‰) values of marine surface water (Surface δ^15^N) and coastal groundwater (CGW δ^15^N) were correlated with δ^15^N (‰) values of both deployed *Ulva* and shore-collected tissues. Spearman’s correlation coefficient is shown as r_s_, the p-value is shown as p, and n represents sample size.

**Fig 2:**
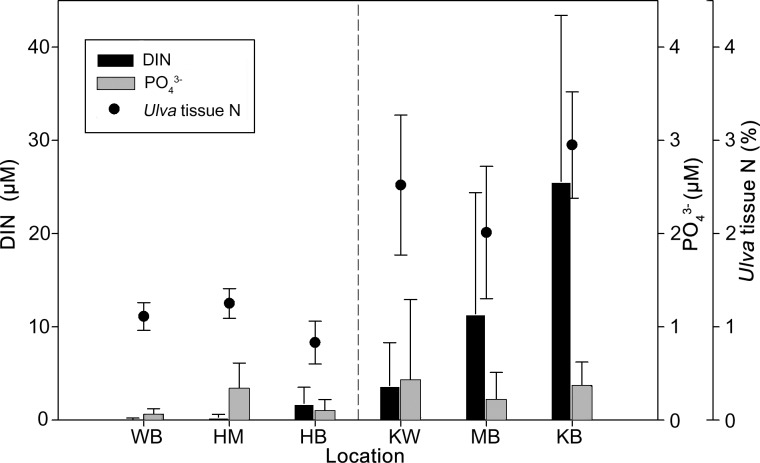
Mean marine surface DIN, phosphate, and deployed *Ulva* tissue N % from all locations. Study locations Waiehu (WB), Honomanū (HM), Honolua (HB), Kahului (KW), Māʻalaea (MB), and Kūʻau (KB) Bays appear in order of increasing marine surface DIN concentration. Mean concentrations of DIN (μM) are shown as dark bars on the primary y-axis. Mean concentration of phosphate (PO_4_^3-^, μM) are shown as light bars and tissue N % values of deployed *Ulva* samples are shown as closed circles on the secondary y-axis. Error bars indicate standard deviation of the mean. The dashed line separates designated Low-N locations (left of line) from High-N locations (right of line).

Significant differences were detected among locations for nutrient concentrations (TDN, DIN, TDP and PO_4_^3-^) of marine surface water (Table 3) and CGW (Table 4) as well as mean values of tissue N parameters (δ^15^N, N %, and C:N) for deployed *Ulva* (Table 5) and shore-collected algae (Table 6). Mean DIN concentrations in marine surface water and tissue N % of deployed *Ulva* were high at Kahului, Māʻalaea, and Kūʻau Bays relative to Waiehu, Honomanū, and Honolua Bays (Fig 2). Based on the detailed characterization of the N concentrations in marine surface water and coastal groundwater at these study locations by Bishop et al. [53] and the results shown in Fig 2, we compared nutrient relationships of locations with the highest N concentrations in deployed *Ulva* tissues and water samples (Kahului, Māʻalaea, and Kūʻau Bays) with those of lowest N concentrations (Waiehu, Honomanū, and Honolua Bays) hereafter referred to as High-N and Low-N locations, respectively. This High-N vs. Low-N designation of location served only as method of result presentation; the following ANOVA comparisons did not use this designation in a statistical manner.

**Table 3:** Average nutrient concentrations of marine surface water.

	Location	n	Salinity	TDN	DIN	TDP	PO_4_^3-^
**Low-N**	**Waiehu**	10	32.2 ± 2.3	4.7 ± 0.8	0.1 ± 0.1	0.00 ± 0.01	0.06 ± 0.06
			**A**	**AC**	**AC**	**A**	**B**
	**Honomanū**	10	32.7 ± 2.8	3.9 ± 0.4	0.3 ± 0.3	0.12 ± 0.14	0.34 ± 0.27
			**A**	**A**	**AC**	**AB**	**A**
	**Honolua**	11	33.7 ± 0.9	8.0 ± 8.3	1.8 ± 1.7	0.09 ± 0.11	0.10 ± 0.12
			**A**	**AC**	**ACD**	**AB**	**AB**
**High-N**	**Kahului**	22	33.4 ± 1.4	11.2 ± 13.6	3.7 ± 4.6	0.17 ± 0.20	0.43 ± 0.86
			**A**	**BC**	**BC**	**AB**	**AB**
	**Māʻalaea**	11	31.7 ± 2.5	15.2 ± 13.3	11.4 ± 13.0	0.15 ± 0.25	0.22 ± 0.29
			**A**	**BC**	**BD**	**AB**	**AB**
	**Kūʻau**	10	32.4 ± 1.8	29.9 ± 18.9	25.6 ± 17.8	0.17 ± 0.13	0.37 ± 0.25
			**A**	**B**	**B**	**B**	**A**

Total dissolved nitrogen (TDN), dissolved inorganic N (DIN), total dissolved phosphorous (TDP), and dissolved phosphate (PO_4_^3-^) values are shown as mean ± standard deviation (μM) for each study location. Significant differences between locations were found for all variables except salinity using Kruskal-Wallis one way ANOVA on ranks (all p < 0.01). Locations that do not share a boldface letter are significantly different (p < 0.05) according to Dunn’s pairwise comparisons on ranks.

**Table 4:** Average nutrient concentrations of CGW.

	Location	n	Salinity	TDN	DIN	TDP	PO_4_^3-^
**Low-N**	**Waiehu**	4	19.7 ± 12.7	5.8 ± 2.1	1.6 ± 1.8	3.09 ± 3.39	3.44 ± 3.39
			**AB**	**A**	**A**	**ABC**	**AC**
	**Honomanū**	5	3.1 ± 5.8	20.3 ± 5.3	8.0 ± 1.9	3.41 ± 1.91	3.87 ± 1.38
			**A**	**AC**	**A**	**ABC**	**AC**
	**Honolua**	6	17.6 ± 13.7	22.7 ± 14.9	17.2 ± 15.3	1.20 ± 0.89	1.27 ± 0.87
			**AB**	**AC**	**AB**	**ABC**	**BC**
**High-N**	**Kahului**	8	6.9 ± 3.3	54.2 ± 48.0	45.3 ± 39.9	0.91 ± 0.57	1.92 ± 0.65
			**AB**	**AC**	**AB**	**AC**	**BC**
	**Māʻalaea**	7	29.2 ± 5.5	45.4 ± 62.0	42.3 ± 62.3	0.83 ± 0.90	1.01 ± 1.03
			**B**	**A**	**A**	**AC**	**BC**
	**Kūʻau**	7	2.9 ± 2.9	525.9 ± 73.9	414.9 ± 37.8	5.25 ± 1.36	4.90 ± 1.06
			**A**	**BC**	**B**	**B**	**A**

Total dissolved nitrogen (TDN), dissolved inorganic N (DIN), total dissolved phosphorus (TDP), and dissolved phosphate (PO_4_^3-^) values are shown as mean ± standard deviation (μM) for each study location with sample size n. Significant differences between locations were found for all variables using Kruskal-Wallis one way ANOVA on ranks (all p < 0.01). Locations that do not share a boldface letter are significantly different (p < 0.05) according to Dunn’s pairwise comparisons on ranks.

**Table 5:** Average nitrogen parameter values of deployed *Ulva* tissues.

		Final	Initial	Final		Initial	Final		Initial	Final	
		n	δ^15^N	δ^15^N		N	N		C:N	C:N	
	Location		(‰)	(‰)		(%)	(%)				
Low-N	WB	10	8.9 ± 0.4	6.5 ± 0.6	**A**	1.7 ± 0.3	1.1 ± 0.2	**AD**	16.9 ± 2.1	24.9 ± 2.7	**A**
	HM	9	8.7 ± 0.4	6.6 ± 0.5	**A**	1.6 ± 0.4	1.3 ± 0.2	**ACE**	18.2 ± 3.1	22.3 ± 2.9	**A**
	HB	8	5.5 ± 0.5	4.4 ± 0.9	**BC**	0.5 ± 0.0	0.8 ± 0.2	**AC**	41.1 ± 3.2	27.5 ± 6.9	**A**
High-N	KW	16	8.9 ± 0.4	7.1 ± 1.4	**A**	1.7 ± 0.3	2.5 ± 0.8	**BF**	16.9 ± 2.1	12.6 ± 4.0	**B**
	MB	10	5.5 ± 0.5	3.9 ± 0.8	**C**	0.5 ± 0.0	2.0 ± 0.7	**BDEF**	41.1 ± 3.2	15.4 ± 6.8	**AB**
	KB	10	8.7 ± 0.4	4.4 ± 0.8	**BC**	1.6 ± 0.4	3.0 ± 0.6	**B**	18.2 ± 3.1	9.9 ± 1.7	**B**

Initial values represent tissue N after preconditioning treatment on day 0 of the deployment. Final values represent tissue N after deployment. Values shown are mean ± SD. One-way Kruskal-Wallis ANOVA detected significant differences among locations for mean *Ulva* tissue δ^15^N (H = 45.603, p < 0.001), N % (H = 45.394, p < 0.001), and tissue C:N (F (H = 42.334, p < 0.001). Locations that do not share a boldface letter are significantly different (p < 0.05) according to Dunn’s test of pairwise comparisons.

**Table 6:** Average nitrogen parameter values of shore-collected algal tissues.

Location	n	δ^15^N (‰)		N %		C:N	
**Kahului Bay**	**36**	**8.5 ± 3.4**	**A**	**3.2 ± 0.8**	**A**	**8.6 ± 1.4**	**AB**
*A*. *spicifera*	11	8.5 **±** 3.4		3.3 **±** 0.5		7.9 **±** 0.6	
*H*. *musciformis*	11	8.5 **±** 3.9		3.6 **±** 0.7		8.2 **±** 1.1	
*Ulva*	13	8.6 **±** 3.1		2.8 **±** 0.9		9.5 **±** 1.7	
**Māʻalaea Bay**	**33**	**4.3 ± 0.7**	**B**	**2.9 ± 1.2**	**A**	**12.1 ± 5.5**	**B**
*Ulva*	33	4.3 ± 0.7		2.9 ± 1.2		12.1 ± 5.5	
**Kūʻau Bay**	**31**	**2.8 ± 0.6**	**C**	**3.5 ± 0.8**	**A**	**8.1 ± 1.8**	**AB**
*A*. *spicifera*	6	2.3 ± 0.9		3.2 ± 0.6		8.0 ± 1.7	
*H*. *musciformis*	12	3.1 ± 0.6		4.1 ± 0.3		7.0 ± 0.4	
*Ulva*	13	2.8 ± 0.4		3.0± 0.7		9.1 ± 2.0	
**Waiehu Bay**	**13**	**4.9 ± 0.4**	**AB**	**1.5 ± 0.5**	**B**	**17.1 ± 5.9**	**C**
*A*. *spicifera*	12	4.9 ± 0.4		1.6 ± 0.4		15.7 ± 2.9	
*Ulva*	1	4.7		0.7		34.5	

Values shown are mean ± standard deviation for locations (boldface) and for individual species by location. One-way Kruskal-Wallis ANOVA detected significant differences among locations for δ^15^N (H = 89.681, p < 0.001), % N (H = 25.192, p < 0.001), and tissue C:N (H = 35.994, p < 0.001). Locations that do not share boldface letter are significantly different (p < 0.05) according to Dunn’s test of pairwise comparisons on ranks. Algal species of interest were not present along the shoreline for collection at Honomanū or Honolua Bays.

### High-N locations

**Fig 3:**
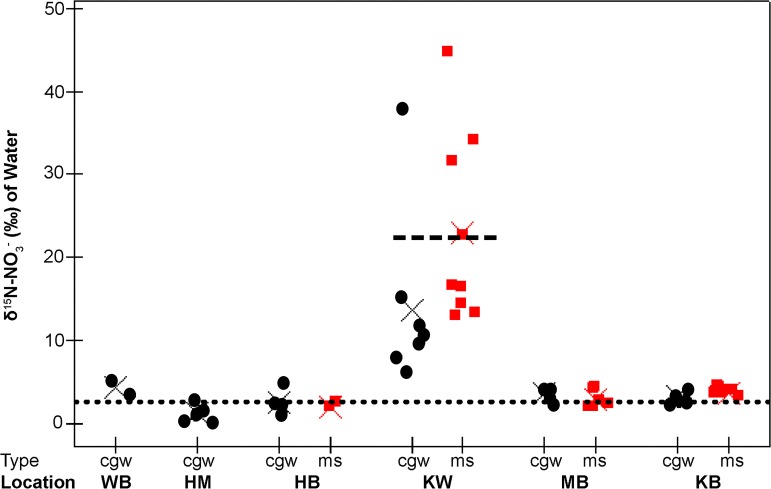
δ^15^N-NO_3_^-^ values of waters for each study location. Coastal groundwater (CGW) sample data is shown as circles and marine surface water sample (ms) data is shown as squares. Study locations Waiehu (WB), Honomanū (HM), Honolua (HB), Kahului (KW), Māʻalaea (MB), and Kūʻau (KB) Bays appear in order of increasing marine surface DIN concentration as shown in Fig 2. The mean δ^15^N value of each category is shown as a cross. The dotted line represents the mean δ^15^N (‰) value of dissolved nitrate (2.3 ± 1.8 ‰) in water sampled from 15 drinking water wells near study locations during 2013 and 2014 [53]. The dashed line represents the δ^15^N value of nitrate in treated wastewater effluent (21.3 ‰) sampled at the Kahului WRF.

Among the locations designated as High-N locations (Kahului, Māʻalaea, and Kūʻau Bays), Kūʻau Bay had the highest concentrations of N in both surface water (TDN = 68.7 μM, DIN = 60.9 μM) and CGW (TDN = 641.9 μM, DIN = 460.8 μM) of all the samples. Mean N (TDN and DIN) concentrations of surface water from Kūʻau Bay were significantly greater than all Low-N locations, but not significantly different from Kahului and Māʻalaea Bays (Table 3). Average values for concentrations of TDN and DIN in CGW at High-N sites (Table 4) were higher than values for surface water but showed similar trends among locations. Values of δ^15^N-NO_3_^-^ were highest at Kahului Bay (Fig 3) in both surface water (mean 23.0 ‰ ± 11.1 ‰) and CGW (mean 13.7 ‰ ± 10.4 ‰). The δ^15^N-NO_3_^-^ value of treated wastewater sampled from the Kahului WRF (21.3 ‰) was similar to the mean value of marine surface water in Kahului Bay (Fig 3). In general, TDP and PO_4_^3-^ were highly variable within and among locations in surface water (Table 3) and CGW (Table 4).

At High-N locations, deployed tissues of *Ulva* attained twice the mean tissue N % and one-half the C:N values relative to Low-N locations (Table 5). Average values at High-N locations ranged from 2.0% ± 0.7% N and 15.4 ± 6.8 C:N at Māʻalaea, to 3.0% ± 0.6% N and 9.9 ± 1.7 C:N at Kūʻau Bay (Table 5). Mean values of *Ulva* tissue N % increased over the deployment period at all High-N locations (Table 5). Final *Ulva* tissue N % and C:N values were generally significantly different between High-N and Low-N locations, but were similar within both designated location groups (Table 5).

**Fig 4:**
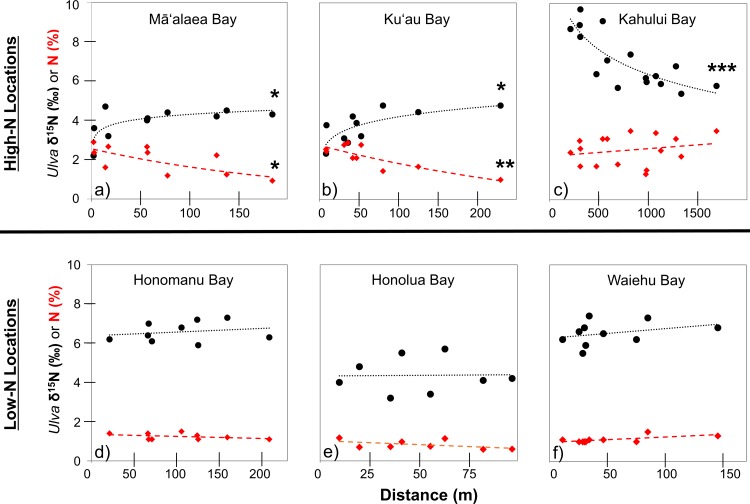
Distance vs. deployed *Ulva* tissue δ^15^N and N %. Final values for deployed *Ulva* tissue δ^15^N (‰) (filled black circles) and N % (filled red diamonds) are shown on the y-axis at all locations: **a)** Māʻalaea Bay, **b)** Kūʻau Bay, **c)** Kahului Bay, **d)** Honomanū Bay, **e)** Honolua Bay, and **f)** Waiehu Bay. The x-axis represents distance (m) from the nearest shoreline except for **c)** where this indicates the linear distance from the Kahului WRF. Black dotted lines represent the regression line calculated for distance vs. *Ulva* tissue δ^15^N and the red, dashed lines represent the regression lines for *Ulva* tissue N %. Significant regressions are indicated as * p < 0.05, ** p < 0.005, *** p< 0.0005. Regression equations and statistical results are shown in S1 Equations.

Distance from shore had a positive relationship with deployed *Ulva* tissue δ^15^N values and a negative relationship with tissue N % at both Māʻalaea (Fig 4A and S1 Fig) and Kuʻau (Fig 4B and S2 Fig) Bays. Deployed *Ulva* tissue N % had a strong negative correlation with tissue δ^15^N values at Kūʻau (r_s_ = -0.91, p < 0.001, n = 10) and Māʻalaea Bays (r_s_ = -0.84, p = 0.002, n = 10). The DIN concentration of marine surface water was positively related to the N % of deployed *Ulva* tissues at Kūʻau (F = 12.8721, r^2^ = 0.62, p = 0.007) and Māʻalaea (F = 8.2962, r^2^ = 0.70, p = 0.014) bays. Shore-collected algae at Kūʻau Bay had lower mean tissue δ^15^N values than all other locations (Table 6). In general, shore-collected and deployed algae at Māʻalaea and Kūʻau Bays had relatively low mean tissue δ^15^N values (2.8 ‰ to 4.4 ‰; Table 5) and relatively high mean tissue N % values (2.0% to 3.5%; Table 6).

**Fig 5:**
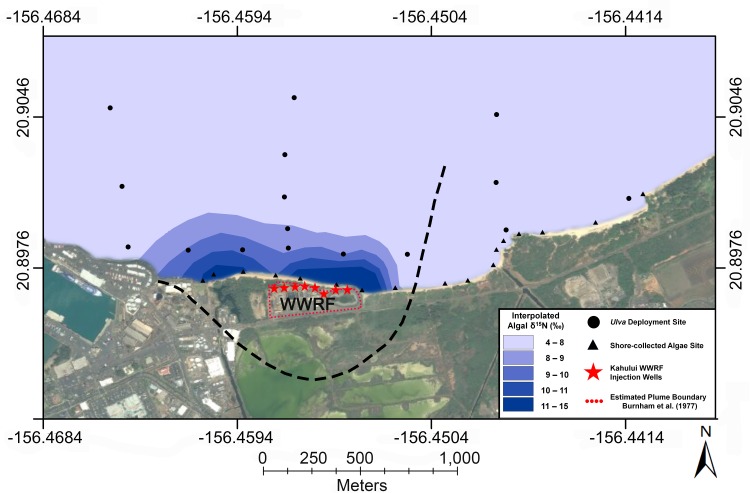
Interpolation of algal tissue δ^15^N values at Kahului Bay. Interpolated δ^15^N values of algal tissues are shown as blue, shaded polygons. Sample sites used in the interpolation are shown as filled, black circles and triangles for deployed *Ulva* and shore-collected algae, respectively. The dashed line represents the two dimensional boundary of wastewater effluent from Kahului WRF injection wells (filled, red stars) as estimated by Burnham et al. [68]. The boundary of the Kahului WRF is shown by the red, dotted line. Satellite imagery was used with permission from Esri (DigitalGlobe, GeoEye, i-cubed, Earthstar Geographics, CNES/Airbus DS, USDA, USGS, AEX, Getmapping, Aerogrid, IGN, IGP, swisstopo, and the GIS User Community; All rights reserved).

A significant negative relationship between distance from the Kahului WRF and deployed *Ulva* tissue δ^15^N values was detected. (Fig 4C, Fig 5 and S3 Fig). A similar trend was found for shore-collected algae in an eastward direction from the WRF (F = 39.8811, r^2^ = 0.71, p < 0.001). In general, *Ulva* deployed nearshore at High-N locations had final δ^15^N values that were similar to mean δ^15^N values of shore-collected algal tissues (S1 Fig, S2 Fig and S3 Fig). Highest tissue δ^15^N values were measured adjacent to the Kahului WRF (S3 Fig) in both deployed *Ulva* (δ^15^N = 9.7 ‰) and shore-collected algae (δ^15^N = 15.3 ‰). Interpolated algal δ^15^N values ≥ 8 ‰ generally fell within the boundary of injected wastewater as estimated by Burnham et al. [68] at the Kahului study location (Fig 5). The region of Kahului Bay that had interpolated algal δ^15^N values ≥ 8 ‰ had an approximate area of 0.31 km^2^.

### Low-N locations

Mean TDN and DIN concentrations of costal surface water and CGW at Low-N locations (Waiehu, Honomanū, and Honolua Bays) were generally an order of magnitude lower and less variable compared to High-N locations (Table 3). TDN and DIN concentrations were not significantly different among Low-N locations in surface water (Table 3) or SGW (Table 4). The lowest values for water δ^15^N-NO_3_^-^ were found in CGW (mean = 1.1 ± 0.7 ‰) at Honomanū Bay.

*Ulva* deployed at Low-N locations yielded tissues with low values for mean N % and high C:N values, with final values ranging from 0.8 ± 0.2% N and 27.5 ± 6.9 C:N at Honolua Bay, to 1.3 ± 0.2% N and 22.3 ± 2.9 C:N at Honomanū Bay (Table 5). Final mean δ^15^N and N % values of deployed *Ulva* were lower than initial values at Honomanū and Waiehu Bays (Table 5). At Honolua Bay, *Ulva* demonstrated a slight increase in mean tissue N % and a slight decrease in mean tissue δ^15^N from initial values (Table 5). Significant relationships between distance from shore and deployed *Ulva* tissue parameters (N % and δ^15^N) were not detected at the Low-N locations Honomanū Bay (Fig 4D and S4 Fig), Honolua Bay (Fig 4E and S5 Fig), or Waiehu Bay (Fig 4F and S6 Fig). Shore-collected algae from Waiehu (Table 6) had slightly lower mean tissue δ^15^N values than deployed *Ulva* samples (Table 5) at this location. Macroalgae were not present for collection near the shoreline at Honomanū or Honolua Bays.

### Benthic analyses: Percent cover and diversity

**Fig 6:**
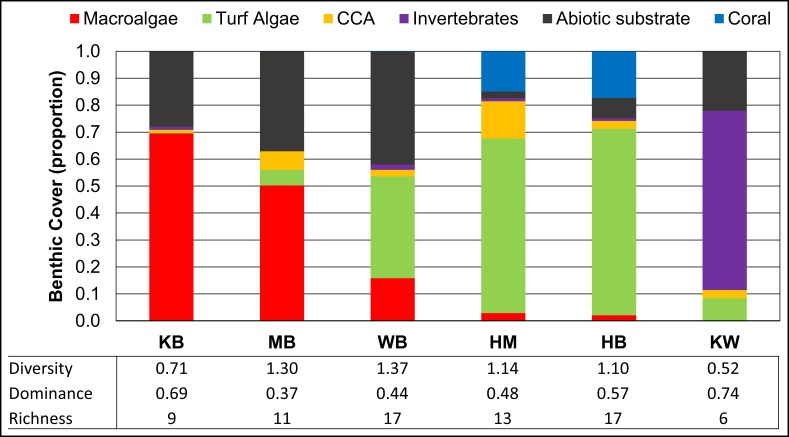
Benthic analyses by location. Benthic cover is shown as the proportion of a benthic type (y-axis) for each location (x-axis). CCA refers to crustose coralline algae. Values for Shannon’s diversity, Simpson’s dominance, and richness are shown for each location. Study locations Kūʻau (KB), Māʻalaea (MB), Waiehu (WB), Honomanū (HM), Honolua (HB), and Kahului (KW) Bays are shown in order of decreasing proportion of macroalgae (red).

Large variation was observed in the benthic assemblage among locations (Fig 6). Kūʻau and Māʻalaea Bays were dominated by fleshy (non-calcified) macroalgae (> 50% cover) with greater than twice the mean percent cover of macroalgae compared to other locations. A significant difference in the proportion of benthic macroalgae among locations was detected (H = 51.45, p < 0.001). Tukey’s pairwise comparisons show Kūʻau and Māʻalaea Bays had a significantly greater proportion of macroalgae than Honomanū Bay (p < 0.01), Honolua Bay (p < 0.01), and Kahului Bay (p < 0.01), but were not significantly different from each other. An inverse relationship between benthic cover of turf algae and macroalgae is apparent in Fig 6, and a negative correlation was detected (r_s_ = -0.64, p < 0.001, n = 60). Corals were only present at Honomanū and Honolua Bays and represented about 15% of benthic surface at these locations (Fig 6). Non-coral invertebrates were rare at most locations except for Kahului Bay, where zoanthids accounted for 50% to 90% of the benthic cover for most transects.

Benthic diversity measures (Shannon’s Diversity, Simpson’s Dominance, and richness) had similar values at Honolua, Honomanū, Waiehu, and Māʻalaea Bays (Fig 6). Kahului and Kūʻau Bays had the highest values for dominance and the lowest values for diversity and richness (Fig 6). The low diversity observed at these locations is driven by the high proportion of zoanthids at Kahului Bay and the siphonous chlorophyte *Derbesia tenuissima*; this species accounted for roughly 60% of benthic surface at Kuʻau Bay. Although *D*. *tenuissima* represented about 35% of the benthic surface at Māʻalaea Bay, at least seven other species of macroalgae were present. One species of non-native macroalgae, *Acanthophora spicifera*, was identified at three of six locations, accounting for about 4% of the benthic surface in Waiehu Bay transects, but less than 0.25% at Kahului and Māʻalaea Bays.

## Discussion

### Reef health and nutrient loading to coastal areas of Maui

Opportunistic and non-native macroalgal blooms constitute a major threat to reef health on the main Hawaiian Islands [22, 23, 30, 32, 33, 69–72]. Although coral cover averaged across 32 long-term monitoring locations in the main Hawaiian Islands has not changed significantly over a 12 year period, results for Maui nonetheless show the highest proportion of impacted sites (44%) with significant losses of live coral [20]. Of the six locations studied here, we find Kūʻau Bay as the most impacted study location because of (1) the relatively high N in algal tissues and in coastal water, (2) the presence of invasive algae at the shoreline, (3) high macroalgal cover, and (4) low benthic diversity. Low algal δ^15^N (‰) values and high tissue N % in this study imply that synthetic fertilizer applied to adjacent sugarcane fields was the most likely source of nutrients to this area. By comparing well water sampled upslope from Kūʻau Bay with CGW, a concurrent study estimates that fertilizer applied to sugarcane and pineapple fields increases coastal groundwater nitrate by 345 μM; this is equivalent to 77% of total nitrate + nitrite (N+N) in CGW [53].

Similar to results from Kūʻau Bay, the combination of low δ^15^N and high N concentrations in *Ulva* tissues and water samples from Māʻalaea Bay suggest that high levels of N loading, from a source with a low δ^15^N value, was present. In a previous study, Dollar et al. [27] reports values for N parameters in algae and water nutrient concentrations that were nearly identical to those reported in this study. Algal tissue δ^15^N values from Māʻalaea samples reported by Dailer et al. [8] show similar trends. Our results concur with those of Dollar et al. [27] and Bishop et al. [53], which indicate that although shallow wastewater injection wells (relatively small facilities associated with individual condominiums at Māʻalaea) may be a source of nutrients in the nearshore zone of Māʻalaea Bay, fertilizer applied to adjacent sugarcane farms is the dominant source of N to this reef. This is reflected in the low δ^15^N values (within the range of nitrate in fertilizer [48]) and the relatively high levels of N in water and algal samples at this location. Bishop et al. [53] estimates that fertilizer applied to sugarcane increased coastal groundwater nitrate by 120 μM of nitrate (39% of total N+N) at Māʻalaea Bay. Dollar et al. [27] estimates wastewater from injection wells accounts for 17% of N input to this bay and concluded that nutrient-rich SGD influences the nearshore reef at Māʻalaea within 100 m of the shoreline. This suggests that fertilizer-derived N that is delivered to reefs via SGD likely plays a major role in supporting the growth and dominance of macroalgae as measured in this study at both Māʻalaea and Kūʻau Bays (Fig 6). Previous studies on West Maui using similar methods suggest that benthic algal blooms may have been supported by fertilizer-enriched groundwater [25, 26, 32]. Macroalgal blooms that cover >70% of the benthic surface have recently been reported at long term monitoring sites adjacent to regions with active sugarcane production on Maui [20, 21]. A long-term reef monitoring site Papaʻula Point (10 m depth), located between Kūʻau Bay and Kahului WRF, has experienced the largest decline in coral cover of all CRAMP sites in Hawaiʻi [20]. A phase shift has occurred at this site [21]; coral cover has decreased from ~ 50% in 1999 to ~ 6% in 2013, while macroalgal cover (mostly *A*. *spicifera*) increased from ~ 25% to 69%, respectively [73].

As shown for other coastal areas impacted by wastewater [3, 8–10, 36, 37, 41–44], algal tissue and water δ^15^N values are effective indicators of wastewater in the nearshore marine environments studied here. A recent study on Maui showed that *Ulva* tissues that were deployed near the Lahaina WRF had a similar ability to detect treated wastewater as a long-term dye tracer test [10, 36]. This study builds on the work of Dailer et al. [8], which detected δ^15^N values as high as 22.3 ‰ in a few algal samples collected adjacent to the Kahului WRF; this is Hawaiʻi’s highest-rate wastewater injection facility [8]. Although δ^15^N values of algal tissues found near the Kahului WRF in this study were lower than those of Dailer et al. [8], they are within the range of values indicative of wastewater [42, 48]. Using both shore-collected and deployed algae, we show that the extent of wastewater effluent from the Kahului WRF in the marine environment (outer boundary of the interpolated algal δ^15^N = 8 ‰, Fig 5) was remarkably similar to the results of numerical models that predicted the subsurface wastewater plume dispersal pathway nearly 30 years ago, prior to the installation of the Kahului WRF [68]. Clear spatial patterns and relatively high δ^15^N values in algal tissues, as well as water parameters for CGW and marine surface water sampled near the Kahului WRF, are consistent with this facility as a source of N to adjacent reefs at Kahului. As seen in this study at Kahului Bay and similar studies, decreases in δ^15^N values with increased distance (from a suspected N point-source) are characteristic of significant wastewater input to marine waters [9, 10, 38, 41–43]. Using naturally occurring isotopic tracers of water (δ^18^O-H_2_O) and salinity, Bishop et al. [53] estimate that the CGW sampled adjacent to the Kahului WRF contains between 26% to 75% wastewater effluent (n = 4).

As a multiple stressor, wastewater contains many co-occurring agents, such as pathogens, endocrine disrupters, heavy metals, pharmaceuticals, personal care products, and industrial chemicals in addition to nutrients, that are known to impact the growth and reproduction of reef-biota [37, 38, 74, 75]. These co-occurring agents have the potential to produce synergistic effects that influence community structure, yet few studies have investigated this [74, 76]. For example, the likelihood of a phase shift from coral to algal dominated reefs (as seen on Maui [20], Oʻahu [33,] and on the Great Barrier Reef [14]) may increase if wastewater-derived pharmaceuticals that reduce herbivore populations co-occur with nutrients that promote algal growth and coral disease [34, 76, 77]. Pharmaceuticals may also inhibit the growth of some benthic biota (such as particular species of macroalgae or invertebrates [76]) while allowing more tolerant organisms to dominate. In this study, the ecological consequences of wastewater discharge from the Kahului WRF are apparent, although the exact reasons for environmental degradation at this site remain unclear. Benthic analyses reveal that the reef adjacent to Kahului WRF had the lowest diversity of all locations in this study (Fig 6), and was almost entirely dominated by colonial zoanthids. This phenomenon has not been reported at any other reef-monitoring site in the main Hawaiian Islands [21], yet increased zoanthid abundance has been related to wastewater discharge at other locations worldwide. Zoanthids were a significant biotic component (> 20% cover) and *Ulva* tissues had higher δ^15^N values at a site impacted by a wastewater outfall in Tobago, West Indies, compared to reference sites [78]. In Puerto Rico, zoanthid dominance on shallow inshore reefs is strongly correlated with *Enterococcus* concentrations, suggesting their dominance under hypertrophic, fecal-polluted conditions [79]. In Bahia, Brazil, zoanthid abundance is linked to increased nutrients associated with wastewater-enriched SGD [13]. In Kāneʻohe Bay, Oʻahu, municipal wastewater input at multiple sites caused a shift in benthic community structure during the early 1970’s from corals to filter feeders such as zoanthids, sponges, and barnacles [29, 33, 80]. In the southern part of Kāneʻohe Bay, extensive and persistent beds of zoanthids replaced scleractinian corals on shallow patch and fringing reefs in the 1960s [81]. Although more research is necessary to determine if a link between the Kahului WRF and the adjacent zoanthids exists, the results of these previous studies suggest injected wastewater effluent may support their growth and dominance in Kahului Bay.

Low-N locations in this study have less anthropogenic disturbance than High-N locations. This is evidenced by relatively low nutrient levels in coastal surface waters, high species richness, the presence of corals, and the low proportion of benthic macroalgae compared to High-N locations. Onshore-offshore gradients in salinity and nutrients, driven by SGD, were observed in surface waters at all locations. At High-N locations, clear onshore-offshore relationships in the N parameters of deployed *Ulva* tissue were observed while no relationship between these parameters and distance from shore was found at Low-N locations. This suggests that the nutrient concentrations and/or the duration of nutrient availability were not high enough at Low-N sites to produce a comparable effect on deployed *Ulva* tissue or benthic community structure.

### SGD as a source of nutrients to marine ecosystems

Nearly all SGD studies indicate that SGD is a significant source of nutrients to coastal ecosystems however, the ecological impact of these nutrients on these systems remains poorly understood [4]. Biological metrics that have been previously included in SGD studies are often associated with phytoplankton [82, 83], which are subject to movement via currents, unlike the sessile benthic macrobiota used in this study. In addition, estimates of SGD flux and nutrient load that are commonly reported in SGD studies [7] may not be appropriate for use as a comparison of nutrient availability to marine biota among some locations. The concentration of bio-available nutrients in nearshore waters, which plays a major role in productivity, is determined by many additional co-dependent factors that vary with location and time such as mixing, water body volume, depth, water residence time, weather, and biotic community.

Correlations among N parameters in CGW, surface water, and algal tissues shown here indicate a clear link between SGD-derived nutrients and reef biota. The results of this work imply that the nutrient concentration of CGW (the SGD endmember) may exert more control on the nutrient concentrations and biological processes in nearshore waters than the volumetric flux of SGD at some locations. For example, the companion study Bishop et al. [53] estimated similar values for scaled freshwater SGD flux (tidal-averaged SGD flux estimates measured at a single point were scaled to the bay scale based on flux measured during a single low tide event at multiple points along the shoreline) at Honolua (4.4 ± 2.5 m^3^ m^-1^ d^-1^) and Kūʻau Bays (3.8 ± 2.1 m^3^ m^-1^ d^-1^). However, the corresponding N+N flux estimate and surface water N+N concentration for Kūʻau Bay was an order of magnitude greater than for Honolua Bay because the N+N concentration in CGW at Kūʻau Bay was an order of magnitude greater. Comparisons between Honolua and Kahului Bays highlight the role of N concentration in CGW for regions with relatively little SGD flux. Although Honolua Bay had more than four times the estimated flux of scaled fresh SGD and twice the N+N load than Kahului Bay [53], the N+N concentration in surface water and in CGW at Kahului Bay was at least two-fold higher.

Although differences in precipitation are expected to impact SGD flux and associated N+N loads among locations and seasons [1, 84], these results suggest that differences in land use and associated nutrient loading to CGW may exert an equal or greater amount of control on the level of available nutrients in the water column than variations SGD flux. For example, Dulai et al. [84] found that maximal SGD rates during the wet season in Kiholo Bay, Hawaiʻi were only 2.3 times greater than the minimum rate of SGD flux, which was measured during the dry season. In contrast, differences in SGD flux and N concentration in CGW among our study locations varied across one order of magnitude and two orders of magnitude, respectively [53]. Because SGD that emerges at many of these locations originates from high-elevation recharge and must travel a long distance to the coast with potential for relatively long travel times [53], seasonal effects on SGD flux may be difficult to detect. To control for seasonal effects, all water samples were taken during the late spring to summer (the end of Hawaiʻi’s wet season) at all locations from 2012 to 2014.

### Algal bioassays: Changes in tissue chemistry across spatial gradients

This study refines the use and interpretation of macroalgal bioassays for environmental studies by showing that (1) with proper selection of the initial values of algal tissue δ^15^N and N %, it is possible to target detection of specific N sources, (2) the presence and type of spatial relationships in both tissue δ^15^N and N % values along onshore-offshore gradients are related to N source and level of N loading, and (3) that the tissues of shore-collected algae reflected values and spatial trends in both deployed algal samples and water quality. Although a number of researchers have used algal tissue δ^15^N values to detect wastewater in coastal areas [8, 9, 13, 27, 37, 46, 67, 85–88], tissue N % was generally not reported. In this study, we showed that tracking changes in both tissue δ^15^N and N % values informed N source identification and level of N loading.

It is often difficult to distinguish fertilizer as a source of N using δ^15^N values alone because many sources have δ^15^N values which span a similar range [48]. We deployed preconditioned *Ulva* tissues with relatively low initial tissue N % and initial δ^15^N values that were slightly higher than those typically reported for synthetic fertilizer and natural soil N [48] to detect N uptake from local sources. The δ^15^N values of these preconditioned tissues ranged from 5.5 ‰ to 8.9 ‰, which were slightly higher than those deployed on West Maui (5 ‰) by Dailer et al. [8]. Samples deployed near shore at Kūʻau and Māʻalaea Bay showed decreases in tissue δ^15^N and increases in N % over the deployment period; uptake of N derived from a source with a low δ^15^N value (i.e., synthetic fertilizer) is the likely explanation. Identical trends in tissue N parameters with distance from shore suggest N-loading via SGD was highest nearshore and decreased with distance from the shoreline at these two locations. These onshore-offshore nutrient gradients are consistent with previous studies on Maui that suggest large amounts of fertilizer-derived N were delivered via SGD to nearshore waters [25–28].

Changes in tissue N % are particularly informative when tissue δ^15^N values remain constant while N % increases. Although the final tissue δ^15^N values of samples deployed near Kahului WRF were similar to pre-deployment values, final tissue N % was greater than twice the initial concentration; this suggests that these samples acquired N from a denitrified source (i.e., treated wastewater effluent). Samples deployed further offshore at Kahului Bay generally had increased tissue N % and decreased tissue δ^15^N compared to initial values; this suggests that final tissue N was a mix of pre-deployment N and uptake of a N source with lower δ^15^N value (i.e., oceanic surface water). In Hawaiʻi, oceanic surface waters (0–150 m) have low δ^15^N values (0 ± 1.0 ‰ to 3.5 ± 0.8 ‰) and DIN concentrations that are at or below the level of detection [89]. We propose that the lack of spatial trends in deployed algal tissue N parameters (δ^15^N and N %) in addition to relatively minimal changes in (or loss of) tissue N %, as observed at Low-N locations in this study (Honolua Bay, Honomanū Bay, and Waiehu Bay), could serve as characteristics of relatively unimpacted coastal ecosystems.

### Conceptual model of nutrient source and loading in Hawaiʻi

**Fig 7:**
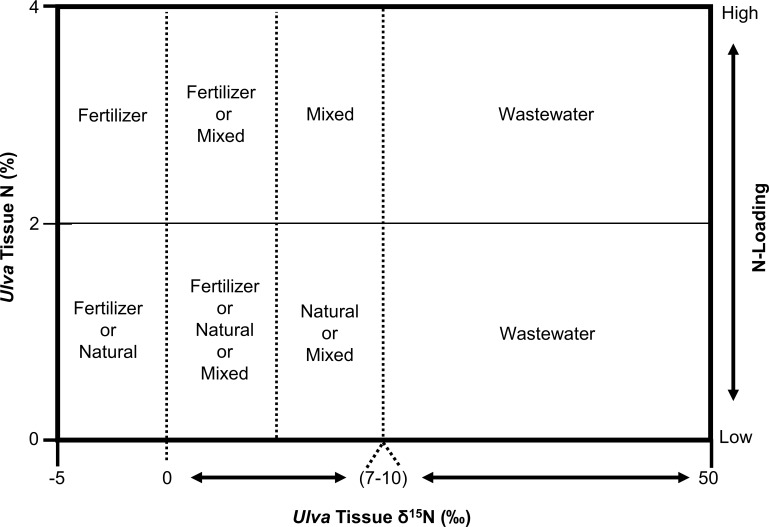
Conceptual model of N loading from potential sources in Hawaiʻi. Values of tissue δ^15^N (‰) and tissue N (%) are shown on the x-axis and y-axis, respectively. Potential N source(s) and the relative amount of N-loading (secondary y-axis) are identified by plotting an *Ulva* sample’s tissue δ^15^N value against its tissue N % value. Natural sources of N include soil, precipitation, and marine surface N. Fertilizer represents N_2_-derived synthetic products of nitrate and/or ammonium. Mixed refers to δ^15^N values which result from N uptake from multiple sources with different δ^15^N signatures. Wastewater refers to denitrified sources of human and animal waste including septic, cesspools, and facilities utilizing primary, secondary, or higher levels of treatment. The range of δ^15^N values for a potential N source is based on reported values reviewed in Kendall et al. [48] and Dailer et al. [8].

Building on the work of Barr et al. [44], we present a conceptual model in Fig 7 to aid in N source identification that integrates both *Ulva* tissue δ^15^N and N % values observed in this and other studies in Hawaiʻi. Unlike the natural baseline range for *Ulva* δ^15^N values of 6.6 ‰ to 8.8 ‰ suggested for New Zealand [44], natural baseline values in this Hawaiʻi model are closer to zero, which reflects a mix of natural terrestrial and marine surface water N sources. In Hawaiʻi, macroalgal tissue δ^15^N values typically range from 0 ‰ to 4 ‰ for samples located at relatively unimpacted sites on Maui [8], Oʻahu [90], Kauaʻi [67], and Hawaiʻi Island [88, 91].

Based on these results, we suggest a value of 2% for *Ulva* tissue N to represent a threshold between locations impacted by relatively high N loading, and relatively unimpacted sites with low N loading in Hawaiʻi. A similar trend was observed in areas with a large N “footprint” across islands in Hawaiʻi [92]. In addition, maximum growth rates were observed for *Ulva lactuca* when tissue N was ≥ 2% in a controlled setting in Hawaiʻi [93]. This conceptual model is intended to aid in the identification of potential N sources and relative amount of loading to Hawaiian coastal ecosystems. It is particularly suited for use with naturally occurring (e.g. shore-collected) *Ulva* samples, in which tissue N parameters reflect N available at a single site. When applying this model to deployed *Ulva* samples, initial values for tissue N parameters should be considered as discussed above.

## Conclusions

Land use can directly impact groundwater quality. However, downstream connections among groundwater quality, submarine groundwater discharge, and coastal ecosystems remain poorly understood. In this study, we demonstrated clear relationships among nutrients in CGW, marine surface waters, and marine plants. These empirical links between marine plants and SGD-derived nutrients allow us to explore a commonly debated yet, rarely investigated hypothesis that SGD is a significant source of nutrients to nearshore marine biota. The results of this study suggest that significant impacts to nearshore biota were observed at locations where CGW was enriched with moderate to high levels of anthropogenic nutrients compared to locations with relatively little CGW enrichment. By comparing the spatial distribution of N in both water and algal samples among locations with various potential N sources, the role agriculture and wastewater have in coastal regions has become more clear; these land use practices are the most likely sources of excess nitrogen in Maui’s coastal waters. Reefs adjacent to sugarcane farms and wastewater injection wells generally had the most macroalgae, low diversity, and the highest N concentrations in algal tissues, coastal groundwater, and marine surface waters. In contrast, macroalgae were generally absent, corals were present, and nutrient concentrations in water were significantly lower at less impacted locations. These results also suggest that the nutrient concentration of CGW and marine surface water may play a greater role in determining reef health than estimates of SGD flux or total daily nutrient loads at some locations. Anthropogenic nutrient enrichment of coastal groundwater and its subsequent delivery via SGD presents a chronic stress to many nearshore ecosystems in tropical settings. These findings led us to consider the large scale and flux of SGD as a significant nexus connecting land use practices, coastal water quality, reef communities, and the substantial implications for resource management.

## Supporting Information

S1 Equations Regression results for δ^15^N (‰) and N % vs. distance at all locations. These results refer to regression lines shown in Fig 4.
(DOCX)

S1 Fig Māʻalaea Bay location map. Sites of *Ulva* deployments, algal shore-collections, marine surface water (MS Water), and coastal groundwater samples (CG Water) are shown as filled circles, triangles, asterisks, and stars, respectively. Symbol colors at these sites indicate sample δ^15^N (‰) values as shown above. Boldface numbers indicate tissue N % values for deployed and shore-collected samples. Satellite imagery was used with permission from Esri (DigitalGlobe, GeoEye, i-cubed, Earthstar Geographics, CNES/Airbus DS, USDA, USGS, AEX, Getmapping, Aerogrid, IGN, IGP, swisstopo, The University of Hawaiʻi School of Ocean and Earth Science and Technology and the GIS User Community; All rights reserved).
(TIF)

S2 Fig Kūʻau Bay location map. Sites of *Ulva* deployments, algal shore-collections, marine surface water (MS Water), and coastal groundwater samples (CG Water) are shown as filled circles, triangles, asterisks, and stars, respectively. Symbol colors at these sites indicate sample δ^15^N (‰) values as shown above. Boldface numbers indicate tissue N % values for deployed and shore-collected samples. Satellite imagery was used with permission from Esri (DigitalGlobe, GeoEye, i-cubed, Earthstar Geographics, CNES/Airbus DS, USDA, USGS, AEX, Getmapping, Aerogrid, IGN, IGP, swisstopo, The University of Hawaiʻi School of Ocean and Earth Science and Technology and the GIS User Community; All rights reserved).
(TIF)

S3 Fig Kahului Bay location map. Sites of *Ulva* deployments, algal shore-collections, marine surface water (MS Water), and coastal groundwater samples (CG Water) are shown as filled circles, triangles, asterisks, and stars, respectively. Symbol colors at these sites indicate sample δ^15^N (‰) values as shown above. Boldface numbers indicate tissue N % values for deployed and shore-collected samples. Satellite imagery was used with permission from Esri, DigitalGlobe, GeoEye, i-cubed, Earthstar Geographics, CNES/Airbus DS, USDA, USGS, AEX, Getmapping, Aerogrid, IGN, IGP, swisstopo, The University of Hawaiʻi School of Ocean and Earth Science and Technology and the GIS User Community; All rights reserved).
(TIF)

S4 Fig Honomanū Bay location map. Sites of *Ulva* deployments and coastal groundwater samples (CG Water) are shown as filled circles and stars, respectively. Symbol colors at these sites indicate sample δ^15^N (‰) values as shown above. Boldface numbers indicate tissue N % values for deployed and shore-collected samples. Satellite imagery was used with permission from Esri (DigitalGlobe, GeoEye, i-cubed, Earthstar Geographics, CNES/Airbus DS, USDA, USGS, AEX, Getmapping, Aerogrid, IGN, IGP, swisstopo, and Technology and the GIS User Community; All rights reserved).
(TIF)

S5 Fig Honolua Bay location map. Sites of *Ulva* deployments and coastal groundwater samples (CG Water) are shown as filled circles and stars, respectively. Symbol colors at these sites indicate sample δ^15^N (‰) values as shown above. Satellite imagery was used with permission from Esri (DigitalGlobe, GeoEye, i-cubed, Earthstar Geographics, CNES/Airbus DS, USDA, USGS, AEX, Getmapping, Aerogrid, IGN, IGP, swisstopo, The University of Hawaiʻi School of Ocean and Earth Science and Technology and the GIS User Community; All rights reserved).
(TIF)

S6 Fig Waiehu Bay location map. Sites of *Ulva* deployments, algal shore-collections, and coastal groundwater samples (CG Water) are shown as filled circles, triangles, and stars, respectively. Symbol colors at these sites indicate sample δ^15^N (‰) values as shown above. Boldface numbers indicate tissue N % values for deployed and shore-collected samples. Satellite imagery was used with permission (Esri, DigitalGlobe, GeoEye, i-cubed, Earthstar Geographics, CNES/Airbus DS, USDA, USGS, AEX, Getmapping, Aerogrid, IGN, IGP, swisstopo, The University of Hawaiʻi School of Ocean and Earth Science and Technology and the GIS User Community; All rights reserved).
(TIF)

S1 Table Spearman’s correlation results for marine surface water samples. The correlation coefficient (r_s_) and p-value (p) are shown for correlations between salinity, silicate (SiO_4_^4-^), total dissolved nitrogen (TDN), dissolved inorganic N (DIN), total dissolved phosphorous (TDP), and dissolved phosphate (PO_4_^3-^). Marine surface water samples from study locations were pooled; n = 74.
(DOCX)

S2 Table Spearman’s correlation results for marine surface water at Honolua Bay. Water samples were collected adjacent to deployment cages at Honolua Bay. The correlation coefficient (r_s_) and p-value (p) are shown for correlations between distance from shore (distance) in meters, salinity, silicate (SiO_4_^4-^), total dissolved nitrogen (TDN), dissolved inorganic N (DIN), total dissolved phosphorous (TDP), and dissolved phosphate (PO_4_^3-^). n = 9.
(DOCX)

S3 Table Spearman’s correlation results for marine surface water at Honomanū Bay. Water samples were collected adjacent to deployment cages at Honomanū Bay. The correlation coefficient (r_s_) and p-value (p) are shown for correlations between distance from shore (distance) in meters, salinity, silicate (SiO_4_^4-^), total dissolved nitrogen (TDN), dissolved inorganic N (DIN), total dissolved phosphorous (TDP), and dissolved phosphate (PO_4_^3-^). n = 9.
(DOCX)

S4 Table Spearman’s correlation results for marine surface water at Kahului Bay. Water samples were collected adjacent to deployment cages at Kahului Bay. The correlation coefficient (r_s_) and p-value (p) are shown for correlations between distance from Kahului WRF (distance) in meters, salinity, silicate (SiO_4_^4-^), total dissolved nitrogen (TDN), dissolved inorganic N (DIN), total dissolved phosphorous (TDP), and dissolved phosphate (PO_4_^3-^). n = 16.
(DOCX)

S5 Table Spearman’s correlation results for marine surface water at Māʻalaea Bay. Water samples were collected adjacent to deployment cages at Māʻalaea Bay. The correlation coefficient (r_s_) and p-value (p) are shown for correlations between distance from shore (distance) in meters, salinity, silicate (SiO_4_^4-^), total dissolved nitrogen (TDN), dissolved inorganic N (DIN), total dissolved phosphorous (TDP), and dissolved phosphate (PO_4_^3-^). n = 10.
(DOCX)

S6 Table Spearman’s correlation results for marine surface water at Kūʻau Bay. Samples were collected adjacent to deployment cages at Kūʻau Bay. The correlation coefficient (r_s_) and p-value (p) are shown for correlations between distance from shore (distance) in meters, salinity, silicate (SiO_4_^4-^), total dissolved nitrogen (TDN), dissolved inorganic N (DIN), total dissolved phosphorous (TDP), and dissolved phosphate (PO_4_^3-^). n = 9.
(DOCX)

S7 Table Spearman’s correlation results for marine surface water at Waiehu Bay. Water samples were collected adjacent to deployment cages at Waiehu Bay. The correlation coefficient (r_s_) and p-value (p) are shown for correlations between distance from shore (distance) in meters, salinity, silicate (SiO_4_^4-^), total dissolved nitrogen (TDN), dissolved inorganic N (DIN), total dissolved phosphorous (TDP), and dissolved phosphate (PO_4_^3-^). n = 10.
(DOCX)

## References

[1] Slomp CP, Van Cappellen P. Nutrient inputs to the coastal ocean through submarine groundwater discharge: Controls and potential impact. J Hydrol. 2004; 295(1–4):64–86.

[2] Fabricius KE. Effects of terrestrial runoff on the ecology of corals and coral reefs: review and synthesis. Mar Pollut Bull. 2005; 50(2):125–46. 10.1016/j.marpolbul.2004.11.028 15737355

[3] Risk MJ. Assessing the effects of sediments and nutrients on coral reefs. Curr Opin Env Sust. 2014; 7:108–17.

[4] Zhang J, Mandal AK. Linkages between submarine groundwater systems and the environment. Curr Opin Env Sust. 2012; 4(2):219–26. 10.1016/j.cosust.2012.03.006.

[5] González FUT, Herrera-Silveira JA, Aguirre-Macedo ML. Water quality variability and eutrophic trends in karstic tropical coastal lagoons of the Yucatán Peninsula. Estuar Coast Shelf Sci. 2008; 76(2):418–30.

[6] Garrison GH, Glenn CR, McMurtry GM. Measurement of submarine groundwater discharge in Kahana Bay, Oʻahu, Hawaiʻi. Limnol Oceanogr. 2003; 48(2):920–8.

[7] Burnett WC, Bokuniewicz H, Huettel M, Moore WS, Taniguchi M. Groundwater and pore water inputs to the coastal zone. Biogeochemistry. 2003; 66(1/2):3–33.

[8] Dailer ML, Knox RS, Smith JE, Napier M, Smith CM. Using δ^15^N values in algal tissue to map locations and potential sources of anthropogenic nutrient inputs on the island of Maui, Hawaiʻi, USA. Mar Pollut Bull. 2010; 60(5):655–71. 10.1016/j.marpolbul.2009.12.021 20070989

[9] Dailer ML, Ramey HL, Saephan S, Smith CM. Algal δ^15^N values detect a wastewater effluent plume in nearshore and offshore surface waters and three-dimensionally model the plume across a coral reef on Maui, Hawaiʻi, USA. Mar Pollut Bull. 2012; 64(2):207–13. 10.1016/j.marpolbul.2011.12.004 22225912

[10] Glenn CR, Whittier RB, Dailer ML, Dulaiova H, El-Kadi AI, Fackrell JK, et al. (University of Hawaiʻi at Mānoa, School of Ocean and Earth Science and Technology, Honolulu, HI). Lahaina groundwater tracer study—Lahania, Maui, Hawaiʻi. Final Report. Honolulu (HI): State of Hawaiʻi Department of Health, US Environmental Protection Agency, US Army Engineer Research and Development Center; 2013. Available: http://www3.epa.gov/region9/water/groundwater/uic-pdfs/lahaina02/lahaina-gw-tracer-study-final-report-june-2013.pdf

[11] Paytan A, Shellenbarger GG, Street JH, Gonneea ME, Davis K, Young MB, et al Submarine groundwater discharge: An important source of new inorganic nitrogen to coral reef ecosystems. Limnol Oceanogr. 2006:343–8.

[12] Naim O. Seasonal responses of a fringing reef community to eutrophication (Reunion Island, Western Indian Ocean). Mar Ecol Prog Ser. 1993; 99:137–51.

[13] Costa JOS, Nimmo M, Attrill MJ. Coastal nutrification in Brazil: A review of the role of nutrient excess on coral reef demise. J South Am Earth Sci. 2008; 25(2):257–70. 10.1016/j.jsames.2007.10.002

[14] McCook LJ. Macroalgae, nutrients and phase shifts on coral reefs: Scientific issues and management consequences for the Great Barrier Reef. Coral Reefs. 1999; 18(4):357–67. 10.1007/s003380050213

[15] Lyons DA, Arvanitidis C, Blight AJ, Chatzinikolaou E, Guy‐Haim T, Kotta J, et al Macroalgal blooms alter community structure and primary productivity in marine ecosystems. Glob Change Biol. 2014; 20:2712–24. 10.1111/gcb.12644 24890042

[16] Lee Y-W, Kim G. Linking groundwater-borne nutrients and dinoflagellate red-tide outbreaks in the southern sea of Korea using a Ra tracer. Estuar Coast Shelf Sci. 2007; 71(1–2):309–17. 10.1016/j.ecss.2006.08.004

[17] LaRoche J, Nuzzi R, Waters R, Wyman K, Falkowski P, Wallace D. Brown tide blooms in Long Island’s coastal waters linked to interannual variability in groundwater flow. Glob Change Biol. 1997; 3(5):397–410.

[18] Paerl HW, Otten TG. Harmful cyanobacterial blooms: causes, consequences, and controls. Microb Ecol. 2013; 54(4):995–1010. 10.1007/s00248-012-0159-y 23314096

[19] Lapointe BE, Clark MW. Nutrient inputs from the watershed and coastal eutrophication in the Florida Keys. Estuaries. 1992; 15(4):465–76.

[20] Rodgers KS, Jokiel PL, Brown EK, Hau S, Sparks R. Over a decade of change in spatial and temporal dynamics of Hawaiian coral reef communities. Pac Sci. 2015; 69(1):1–13.

[21] Walsh W, Sparks R, Barnett C, Couch C, Cotton S, White D, et al. (Hawaiʻi Department of Land and Natural Resources, Division of Aquatic Resources, Honolulu, HI). Long-term monitoring of coral reefs of the main Hawaiian islands. Final Report. Honolulu (HI): NOAA Coral Reef Conservation Program; 2010. Dec. Report No.: NA06NOS4260113. Available: https://dlnr.hawaii.gov/dar/files/2014/04/NOAA_2013_WHi_-Mon_-Rep.pdf

[22] Friedlander A, Aeby G, Brainard R, Brown E, Chaston K, Clark A, et al. The state of coral reef ecosystems of the main Hawaiian Islands. In: Waddell JE, Clark AM, editors. The state of coral reef ecosystems of the United States and Pacific freely associated states NOAA Technical Memorandum NOS NCCOS 73 NOAA/NCCOS Center for Coastal Monitoring and Assessment’s Biogeography Team. Silver Spring, Maryland: NOAA; 2008. p. 219–61.

[23] Smith JE, Hunter CL, Smith CM. Distribution and reproductive characteristics of nonindigenous and invasive marine algae in the Hawaiian Islands. Pac Sci. 2002; 56(3):299–315.

[24] Van Beukering P, Cesar HS. Ecological economic modeling of coral reefs: evaluating tourist overuse at Hanauma Bay and algae blooms at the Kihei Coast, Hawaiʻi. Pac Sci. 2004; 58(2):243–60.

[25] Soicher A, Peterson F. Terrestrial nutrient and sediment fluxes to the coastal waters of west Maui, Hawaiʻi. Pac Sci. 1997; 51(3):221–32.

[26] Dollar S, Andrews C (University of Hawaiʻi at Mānoa, School of Ocean and Earth Science and Technology, Honolulu, HI). Algal blooms off west Maui: Assessing the causal linkages between land and the coast ocean. Final Report. Honolulu (HI): US Dept of Commerse, NOAA Coastal Ocean Program Office, University of Hawaiʻi SeaGrant College Program; 1997. Mar.

[27] Dollar S, Atkinson M, Hochberg E, Nance T (Marine Research Consultants Inc., Honolulu, HI). An evaluation of causal factors affecting coral reef community structure in Mā‘alaea Bay, Maui, Hawaiʻi. Final Report. Honolulu (HI): County of Maui, Department of Finance; 2011. Jun. Available: http://www.co.maui.hi.us/DocumentCenter/View/83262

[28] Laws E, Brown D, Peace C. Coastal water quality in the Kihei and Lahaina districts of the island of Maui, Hawaiian Islands. Impacts from physical habitat and groundwater seepage: implications for water quality standards. Int J Environ Pollut. 2004; 22(5):531–46.

[29] Hunter CL, Evans CW. Coral reefs in Kāne‘ohe Bay, Hawaiʻi: two centuries of western influence and two decades of data. Bull Mar Sci. 1995; 57(2):501–15.

[30] Smith JE, Hunter CL, Conklin EJ, Most R, Sauvage T, Squair C, et al Ecology of the invasive red alga *Gracilaria salicornia* (Rhodophyta) on Oʻahu, Hawaiʻi. Pac Sci. 2004; 58(2):325–43.

[31] Lirman D. Competition between macroalgae and corals: effects of herbivore exclusion and increased algal biomass on coral survivorship and growth. Coral Reefs. 2001; 19(4):392–9. 10.1007/s003380000125

[32] Smith JE, Runcie JW, Smith CM. Characterization of a large-scale ephemeral bloom of the green alga *Cladophora sericea* on the coral reefs of west Maui, Hawaiʻi. Mar Ecol Prog Ser. 2005; 302:77–91.

[33] Smith SV, Kimmerer WJ, Laws EA, Brock RE, Walsh TW. Kāneʻohe Bay sewage diversion experiment: Perspectives on ecosystem responses to nutritional perturbabation. Pac Sci. 1981; 35:279–397.

[34] McCook L, Jompa J, Diaz-Pulido G. Competition between corals and algae on coral reefs: a review of evidence and mechanisms. Coral Reefs. 2001; 19(4):400–17. 10.1007/s003380000129

[35] Smith JE, Shaw M, Edwards RA, Obura D, Pantos O, Sala E, et al Indirect effects of algae on coral: algae‐mediated, microbe‐induced coral mortality. Ecol Lett. 2006; 9(7):835–45. 10.1111/j.1461-0248.2006.00937.x 16796574

[36] Glenn CR, Whittier RB, Dailer ML, Dulaiova H, El-Kadi AI, Fackrell JK, et al. (University of Hawaiʻi at Mānoa, School of Ocean and Earth Science and Technology, Honolulu, HI). Lahaina groundwater tracer study—Lahaina, Maui, Hawaiʻi. Final Interim Report. Honolulu (HI): 2012. Nov. State of Hawaiʻi Department of Health, U.S. Environmental Protection Agency, U.S. Army Engineer Research and Development Center. Available: http://www3.epa.gov/region9/water/groundwater/uic-pdfs/lahaina02/lahaina-final-interim-report.pdf

[37] Hunt CD, Rosa SN (U.S. Geological Survey, Honolulu, HI). A multitracer approach to detecting wastewater plumes from municipal injection wells in nearshore marine waters at Kihei and Lahaina, Maui, Hawaiʻi. Final Report. Honolulu (HI): 2009. Report No.: 2009–5253. Available: http://pubs.usgs.gov/sir/2009/5253/sir2009-5253.pdf

[38] Yoshioka RM, Kim CJS, Tracy AM, Most R, Harvell CD. Linking sewage pollution and water quality to spatial patterns of *Porites lobata* growth anomalies in Puako, Hawaii. Mar Pollut Bull. 2016; 104(1–2):313–21. 10.1016/j.marpolbul.2016.01.002 26781454

[39] Moynihan MA, Baker DM, Mmochi AJ. Isotopic and microbial indicators of sewage pollution from Stone Town, Zanzibar, Tanzania. Mar Pollut Bull. 2012; 64(7):1348–55. 10.1016/j.marpolbul.2012.05.001 22682879

[40] Lapointe BE. Nutrient thresholds for bottom-up control of macroalgal blooms on coral reefs in Jamaica and southeast Florida. Limnol Oceanogr. 1997; 42(5):1119–31.

[41] Savage C, Elmgren R. Macroalgal (*Fucus vesiculosus*) δ^*15*^N values trace decrease in sewage influence. Ecol Appl. 2004; 14(2):517–26.

[42] Costanzo SD, Udy J, Longstaff B, Jones A. Using nitrogen stable isotope ratios δ^15^N of macroalgae to determine the effectiveness of sewage upgrades: changes in the extent of sewage plumes over four years in Moreton Bay, Australia. Mar Pollut Bull. 2005; 51(1–4):212–7. 10.1016/j.marpolbul.2004.10.018 15757722

[43] Risk MJ, Lapointe BE, Sherwood OA, Bedford BJ. The use of δ^15^N in assessing sewage stress on coral reefs. Mar Pollut Bull. 2009; 58(6):793–802. 10.1016/j.marpolbul.2009.02.008 19286230

[44] Barr NG, Dudley BD, Rogers KM, Cornelisen CD. Broad-scale patterns of tissue-δ^15^N and tissue-N indices in frondose *Ulva* spp.; Developing a national baseline indicator of nitrogen-loading for coastal New Zealand. Mar Pollut Bull. 2013; 67(1–2):203–16. 10.1016/j.marpolbul.2012.11.033 23260648

[45] Fertig B, Carruthers TJB, Dennison WC, Jones AB, Pantus F, Longstaff B. Oyster and macroalgae bioindicators detect elevated δ^15^N in Maryland’s coastal bays. Estuar Coasts. 2009; 32(4):773–86. 10.1007/s12237-009-9148-x

[46] Umezawa Y, Miyajima T, Yamamuro M, Kayanne H, Koike I. Fine-scale mapping of land-derived nitrogen in coral reefs by δ^15^N in macroalgae. Limnol Oceanogr. 2002; 47(5):1405–16.

[47] Heaton T. Isotopic studies of nitrogen pollution in the hydrosphere and atmosphere: A review. Chem Geol. 1986; 59:87–102.

[48] Kendall C, Elliott EM, Wankel SD. Tracing anthropogenic inputs of nitrogen to ecosystems In: Michener RH, Lajtha K, editors. Stable isotopes in ecology and environmental science. 2 Malden, Massachusetts: John Wiley & Sons; 2007 p. 375–449.

[49] Owens NJ. Natural variations in ^15^N in the marine environment. Adv Mar Biol. 1987; 24:389–451.

[50] Atkinson M, Smith S. C:N:P ratios of benthic marine plants. Limnol Oceanogr. 1983; 28:568–74.

[51] Teichberg M, Fox SE, Olsen YS, Valiela I, Martinetto P, Iribarne O, et al Eutrophication and macroalgal blooms in temperate and tropical coastal waters: Nutrient enrichment experiments with *Ulva* spp. Glob Change Biol. 2010; 16(9):2624–37. 10.1111/j.1365-2486.2009.02108.x

[52] Fong P, Fong JJ, Fong CR. Growth, nutrient storage, and release of dissolved organic nitrogen by *Enteromorpha intestinalis* in response to pulses of nitrogen and phosphorus. Aquat Bot. 2004; 78(1):83–95.

[53] Bishop JM, Amato DW, Glenn CR, Dulai H. Effect of land use and groundwater flow path on submarine groundwater discharge nutrient flux. J Hydrol Reg Stud. 2015 10.1016/j.ejrh.2015.10.008

[54] Whittier RB, El-Kadi AI (University of Hawai‘i at Mānoa, School of Ocean and Earth Science Technology, Honolulu, HI). Human health and environmental risk ranking of on-site sewage disposal systems for the Hawaiian islands of Kauaʻi, Molokaʻi, Maui, and Hawaiʻi. Honolulu (HI): State of Hawaiʻi, Safe Drinking Water Branch; 2014.

[55] Friedlander AM, Brown EK, Monaco ME. Coupling ecology and GIS to evaluate efficacy of marine protected areas in Hawaiʻi. Ecol Appl. 2007; 17(3):715–30. 17494391 10.1890/06-0536

[56] Johnson AG, Glenn CR, Burnett WC, Peterson RN, Lucey PG. Aerial infrared imaging reveals large nutrient-rich groundwater inputs to the ocean. Geophys Res Lett. 2008; 35(15):L15606 10.1029/2008gl034574

[57] Sweeney R, Liu K, Kaplan I. Oceanic nitrogen isotopes and their uses in determining the source of sedimentary nitrogen In: Robinson BW, editor. Stable isotopes in the earth sciences 9 Lower Hutt, New Zealand: New Zealand DSIR Bulletins; 1978 p. 9–26.

[58] O’Kelly CJ, Kurihara A, Shipley TC, Sherwood AR. Molecular assessment of *Ulva* spp. (Ulvophyceae, Chlorophyta) in the Hawaiian Islands. J Phycol. 2010; 46(4):728–35. 10.1111/j.1529-8817.2010.00860.x

[59] Sigman D, Casciotti K, Andreani M, Barford C, Galanter M, Böhlke J. A bacterial method for the nitrogen isotopic analysis of nitrate in seawater and freshwater. Anal Chem. 2001; 73(17):4145–53. 11569803 10.1021/ac010088e

[60] Granger J, Sigman DM, Prokopenko MG, Lehmann MF, Tortell PD. A method for nitrite removal in nitrate N and O isotope analyses. Limnol Oceanogr Methods. 2006; 4:205–12.

[61] Jokiel PL. CRAMP rapid assessment: Benthic protocols 2008 [cited 3/1/2014]. In: Coral reef assessment and monitoring program [Internet]. Hawaiʻi: Hawaiʻi Institute of Marine Biology Available: http://cramp.wcc.hawaii.edu/Rapid_Assessment_Files/RA_benthic_protocol.htm

[62] Brown EK, Cox E, Jokiel P, Rodgers KuS, Smith WR, Tissot BN, et al Development of benthic sampling methods for the Coral Reef Assessment and Monitoring Program (CRAMP) in Hawaiʻi. Pac Sci. 2004; 58(2):145–58.

[63] Bird CE. PhotoGrid Beta 1.0. Honolulu, Hawai‘i 2001.

[64] Shannon CE. A mathematical theory of communication. Bell Syst Tech J. 1948; 27(4):623–56. 10.1002/j.1538-7305.1948.tb00917.x

[65] Simpson EH. Measurement of diversity. Nature. 1949; 163:688.

[66] State of Hawaiʻi Office of Planning. Hawaiʻi statewide GIS program 2014 [cited 8/28/2014]. In: State of Hawaiʻi Office of Planning Honolulu, Hawaiʻi: State of Hawaiʻi Available: http://planning.hawaii.gov/gis/download-gis-data

[67] Derse E, Knee KL, Wankel SD, Kendall C, Berg CJ, Paytan A. Identifying sources of nitrogen to Hanalei Bay, Kaua‘i, utilizing the nitrogen isotope signature of macroalgae. Environ Sci Technol. 2007; 41(15):5217–23. 10.1021/es0700449 17822082

[68] Burnham WL, Larson SP, Cooper HH (U.S. Geological Survey, Honolulu, HI). Distribution of injected wastewater in the saline lava aquifer, Wailuku-Kahului wastewater treatment facility, Kahului, Maui, Hawaiʻi. Final Report. Honolulu (HI): 1977. Report No.: 77–469. Available: http://books.google.com/books?id=MtzltwAACAAJ

[69] Nishimura N. Introduced macroalgae in Hawaiian waters, with special reference to the invasive red alga, Gracilaria salicornia (C. Agardh) Dawson M.S. Thesis. University of Hawaiʻi at Mānoa. 2000.

[70] Smith JE. Invasive macroalgae on tropical reefs: Impacts, interactions, mechanisms and management. J Phycol. 2003; 39(s1):53 10.1111/j.0022-3646.2003.03906001_153.x

[71] Conklin EJ, Smith JE. Abundance and spread of the invasive red algae, *Kappaphycus* spp., in Kāne’ohe Bay, Hawai’i and an experimental assessment of management options. Biol Invasions. 2005; 7(6):1029–39. 10.1007/s10530-004-3125-x

[72] Martinez JA, Smith CM, Richmond RH. Invasive algal mats degrade coral reef physical habitat quality. Estuar Coast Shelf Sci. 2012; 99:42–9.

[73] DLNR-DAR, UH-CRAMP. Long-term benthic cover data set of the main Hawaiian Islands. Department of Land and Natural Resources- Division of Aquatic Resources (DLNR-DAR) and University of Hawaiʻi—Coral Reef Assessement and Monitoring Program (UH-CRAMP). Honolulu, Hawaiʻi: NOAA; 2013.

[74] Wear SL, Thurber RV. Sewage pollution: mitigation is key for coral reef stewardship. Ann N Y Acad Sci. 2015; 1355(1):15–30. 10.1111/nyas.12785 25959987 PMC4690507

[75] Meador JP, Yeh A, Young G, Gallagher EP. Contaminants of emerging concern in a large temperate estuary. Environ Pollut. 2016; 213:254–67. 10.1016/j.envpol.2016.01.088 26907702 PMC5509463

[76] Prichard E, Granek EF. Effects of pharmaceuticals and personal care products on marine organisms: from single-species studies to an ecosystem-based approach. Environmental Science and Pollution Research. 2016:1–20. 10.1007/s11356-016-7282-0 27617334

[77] Gaw S, Thomas KV, Hutchinson TH. Sources, impacts and trends of pharmaceuticals in the marine and coastal environment. Philosophical Transactions of the Royal Society B: Biological Sciences. 2014; 369(1656). 10.1098/rstb.2013.0572 25405962 PMC4213585

[78] Lapointe BE, Langton R, Bedford BJ, Potts AC, Day O, Hu C. Land-based nutrient enrichment of the Buccoo Reef Complex and fringing coral reefs of Tobago, West Indies. Mar Pollut Bull. 2010; 60(3):334–43. 10.1016/j.marpolbul.2009.10.020 20034641

[79] Hernández-Delgado E, Sandoz B, Bonkosky M, Norat-Ramírez J, Mattei H, editors. Impacts of non-point source sewage pollution on Elkhorn coral, Acropora palmata (Lamarck), assemblages of the southwestern Puerto Rico shelf. 11th International Coral Reef Symposium; 2008; Fort Lauderdale, FL.

[80] Laws EA. Aquatic pollution: An introductory text New York, New York: John Wiley & Sons; 2000. 672 p.

[81] Walsh GE, Bowers RL. A review of Hawaiian zoanthids with descriptions of three new species. Zool J Linn Soc. 1971; 50(2):161–80. 10.1111/j.1096-3642.1971.tb00757.x

[82] Welti N, Gale D, Hayes M, Kumar A, Gasparon M, Gibbes B, et al Intertidal diatom communities reflect patchiness in groundwater discharge. Estuar Coast Shelf Sci. 2015; 163:116–24.

[83] Lecher AL, Mackey K, Kudela R, Ryan J, Fisher A, Murray J, et al Nutrient loading through submarine groundwater discharge and phytoplankton growth in Monterey Bay, CA. Environ Sci Technol. 2015; 49(11):6665–73. 10.1021/acs.est.5b00909 25988258

[84] Dulai H, Kamenik J, Waters CA, Kennedy J, Babinec J, Jolly J, et al Autonomous long-term gamma-spectrometric monitoring of submarine groundwater discharge trends in Hawaiʻi. Journal of Radioanalytical and Nuclear Chemistry. 2016; 307(3):1865–70. 10.1007/s10967-015-4580-9

[85] Costanzo SD, O'Donohue MJ, Dennison WC. *Gracilaria edulis* (Rhodophyta) as a biological indicator of pulsed nutrients in oligotrophic waters. J Phycol. 2000; 36(4):680–5. 10.1046/j.1529-8817.2000.99180.x 29542151

[86] Riera P, Stal LJ, Nieuwenhuize J. Heavy δ^15^N in intertidal benthic algae and invertebrates in the Scheldt Estuary (The Netherlands): effect of river nitrogen inputs. Estuar Coast Shelf Sci. 2000; 51(3):365–72. 10.1006/ecss.2000.0684

[87] Cole ML, Kroeger KD, McClelland JW, Valiela I. Macrophytes as indicators of land‐derived wastewater: Application of a δ^15^N method in aquatic systems. Water Resour Res. 2005; 41(1):W01014 10.1029/2004WR003269

[88] Dailer M, Smith C, Glenn C (University of Hawaiʻi at Mānoa, Honolulu, HI). Preventing the introduction and spread of nutrient driven invasive algal blooms and coral reef degradation in West Hawaiʻi. Final Report. Honolulu (HI): Hawaiʻi Coral Reef Initiative; 2013.

[89] Casciotti KL, Trull TW, Glover DM, Davies D. Constraints on nitrogen cycling at the subtropical north pacific station ALOHA from isotopic measurements of nitrate and particulate nitrogen. Deep-Sea Res Pt II. 2008; 55(14–15):1661–72. 10.1016/j.dsr2.2008.04.017

[90] Cox TE, Smith CM, Popp BN, Foster MS, Abbott IA. Can stormwater be detected by algae in an urban reef in Hawai‘i? Mar Pollut Bull. 2013; 71(1):92–100. 23643406 10.1016/j.marpolbul.2013.03.030

[91] Kim CJS, Yoshioka RM, Tracy AM, Harvell DC (Cornell University, Ithaca, NY). Linking sewage pollution and water quality to spatial patterns of Porites growth anomalies in Puakō, Hawai‘i. Final Report. Ithaca (NY): Puakō Community Association; 2014. Sept. Available: http://coral.org/wordpress/wp-content/uploads/2014/09/FINAL-for-PCA-Sewage-and-Porites-growth-anomalies.pdf

[92] Van Houtan KS, Smith CM, Dailer ML, Kawachi M. Eutrophication and the dietary promotion of sea turtle tumors. PeerJ. 2014; 2:e602 10.7717/peerj.602 25289187 PMC4184234

[93] Dailer ML, Smith JE, Smith CM. Responses of bloom forming and non-bloom forming macroalgae to nutrient enrichment in Hawai‘i, USA. Harmful Algae. 2012; 17:111–25. 10.1016/j.hal.2012.03.008

