# Supplementary material for: Impact of Submarine Groundwater Discharge on Marine Water Quality and Reef Biota of Maui
Source: PLoS One. 2016 Nov 3;11(11):e0165825. doi: 10.1371/journal.pone.0165825 (PMC5094668; doi:10.1371/journal.pone.0165825)
Supplement: S1 Equations — These results refer to regression lines shown in Fig 4. (DOCX) [file pone.0165825.s001.docx]

**S1 Equations.** **Regression results for δ^15^N (‰) and N % vs. distance at all locations.**

These results refer to regression lines shown in Fig 4.

**a)** Māʻalaea Bay. δ^15^N (‰) = 2.7411 + 0.3391*ln(X), F = 8.5348, R^2^= 0.52, p= 0.0192.

N % = 2.5598*e^(-0.0039*X)^, F = 6.6089, r^2^ = 0.45, p= 0.0331.

**b)** Kūʻau Bay. δ^15^N (‰) = 2.7188 *X^0.1244^, F = 9.2524, r^2^ = 0.54, p = 0.0160.

N % = 3.5061*e^(-0.0028*X)^, F = 20.0566, r^2^= 0.71, p< 0.0021.

**c)** Kahului Bay. δ^15^N (‰) = 18.9551 – 1.8191*ln(X), F = 27.3428, r^2^ = 0.66, p = 0.0001.

N % = 2.2339 + 0.0004*X, F = 0.6603, r^2^ = 0.05, p = 0.4300.

**d)** Honomanū Bay. δ^15^N (‰) = 6.3815 + 0.0019(X), F = 0.3136, r^2^ = 0.05, p = 0.5930.

N % = 1.3995 - 0.0014, F = 2.2814, r^2^= 0.25, p = 0.1747).

**e)** Waiehu Bay. δ^15^N (‰) = 6.2712 + 0.0049(X), F = 0.3136, r^2^ = 0.11, p = 0.3553.

N % = 0.9837 + 0.0024, F = 5.1916, r^2^ = 0.39, p = 0.0522).

**f)** Honolua Bay. δ^15^N (‰) = 4.3292 + 0.0007(X), F = 0.0028, r^2^ = 0.00, p = 0.9594.

N % = 1.0301 - 0.0040, F = 2.0568, r^2^ = 0.26, p = 0.2015).
