# Supplementary figures and images for: Impact of Submarine Groundwater Discharge on Marine Water Quality and Reef Biota of Maui
Source: PLoS One. 2016 Nov 3;11(11):e0165825. doi: 10.1371/journal.pone.0165825 (PMC5094668; doi:10.1371/journal.pone.0165825)

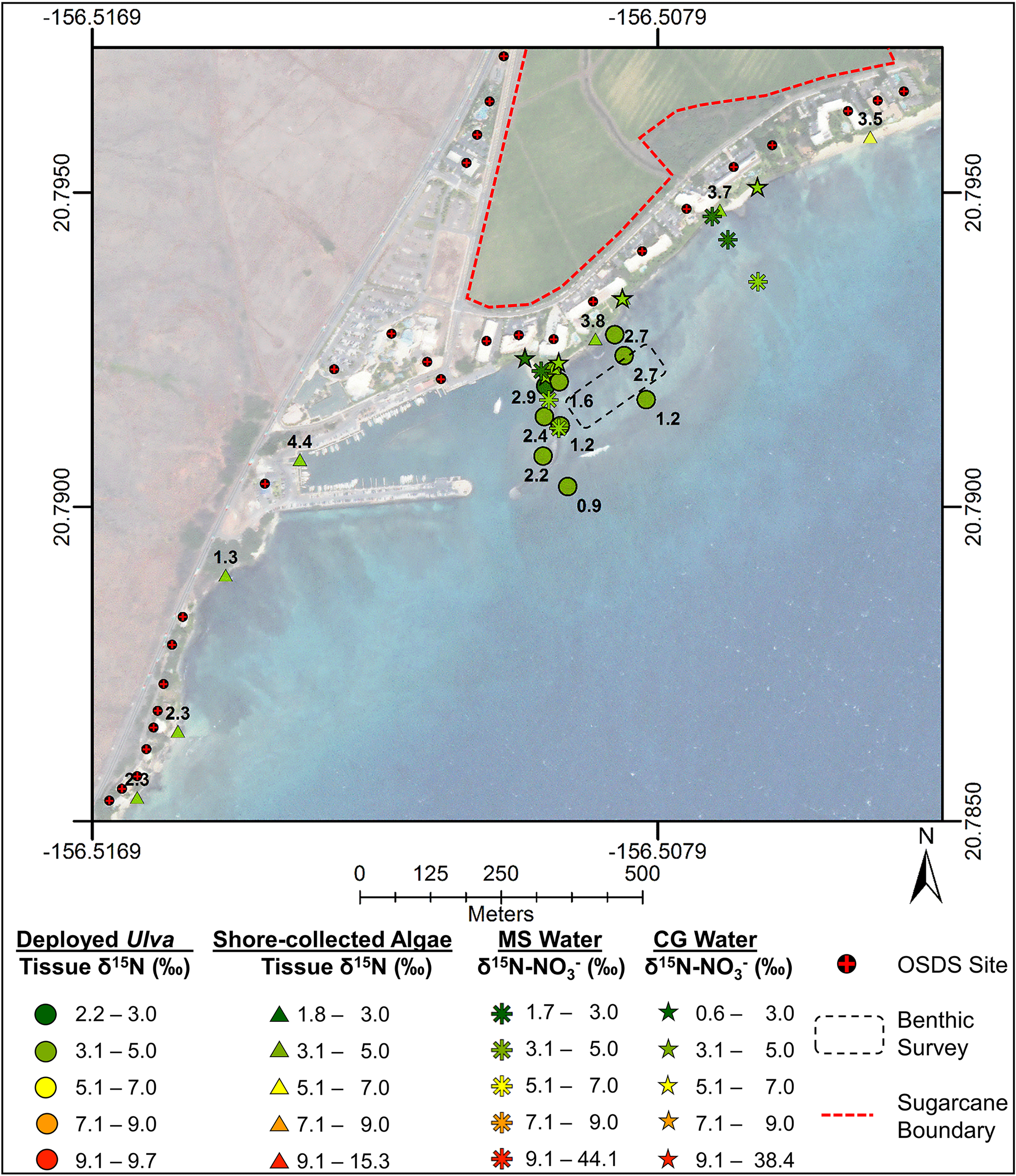

Supplement: S1 Fig — Sites of Ulva deployments, algal shore-collections, marine surface water (MS Water), and coastal groundwater samples (CG Water) are shown as filled circles, triangles, asterisks, and stars, respectively. Symbol colors at these sites indicate sample δ15N (‰) values as shown above. Boldface numbers indicate tissue N % values for deployed and shore-collected samples. Satellite imagery was used with permission from Esri (DigitalGlobe, GeoEye, i-cubed, Earthstar Geographics, CNES/Airbus DS, USDA, USGS, AEX, Getmapping, Aerogrid, IGN, IGP, swisstopo, The University of Hawaiʻi School of Ocean and Earth Science and Technology and the GIS User Community; All rights reserved). (TIF) [file pone.0165825.s002.tif]

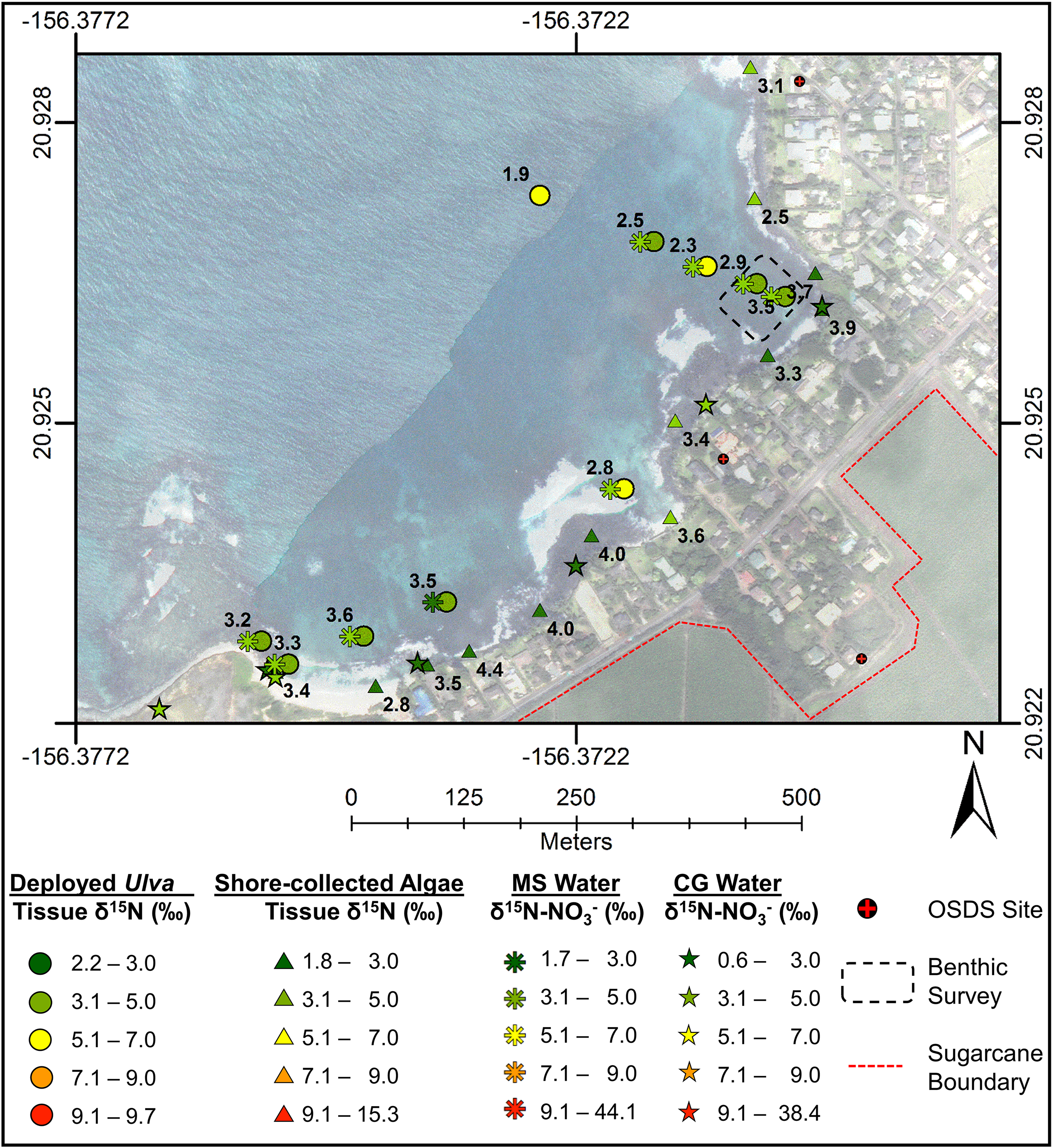

Supplement: S2 Fig — Sites of Ulva deployments, algal shore-collections, marine surface water (MS Water), and coastal groundwater samples (CG Water) are shown as filled circles, triangles, asterisks, and stars, respectively. Symbol colors at these sites indicate sample δ15N (‰) values as shown above. Boldface numbers indicate tissue N % values for deployed and shore-collected samples. Satellite imagery was used with permission from Esri (DigitalGlobe, GeoEye, i-cubed, Earthstar Geographics, CNES/Airbus DS, USDA, USGS, AEX, Getmapping, Aerogrid, IGN, IGP, swisstopo, The University of Hawaiʻi School of Ocean and Earth Science and Technology and the GIS User Community; All rights reserved). (TIF) [file pone.0165825.s003.tif]

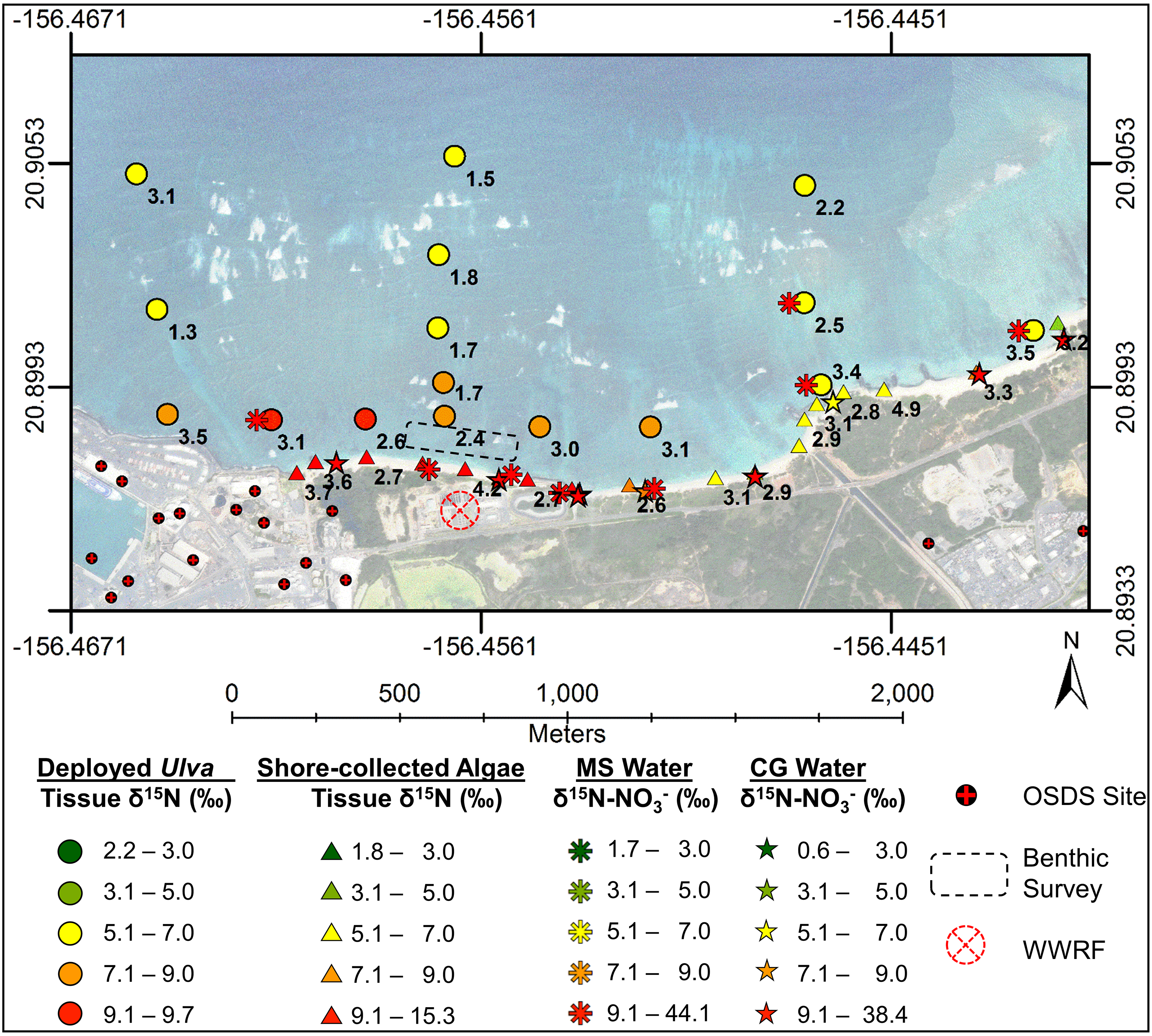

Supplement: S3 Fig — Sites of Ulva deployments, algal shore-collections, marine surface water (MS Water), and coastal groundwater samples (CG Water) are shown as filled circles, triangles, asterisks, and stars, respectively. Symbol colors at these sites indicate sample δ15N (‰) values as shown above. Boldface numbers indicate tissue N % values for deployed and shore-collected samples. Satellite imagery was used with permission from Esri, DigitalGlobe, GeoEye, i-cubed, Earthstar Geographics, CNES/Airbus DS, USDA, USGS, AEX, Getmapping, Aerogrid, IGN, IGP, swisstopo, The University of Hawaiʻi School of Ocean and Earth Science and Technology and the GIS User Community; All rights reserved). (TIF) [file pone.0165825.s004.tif]

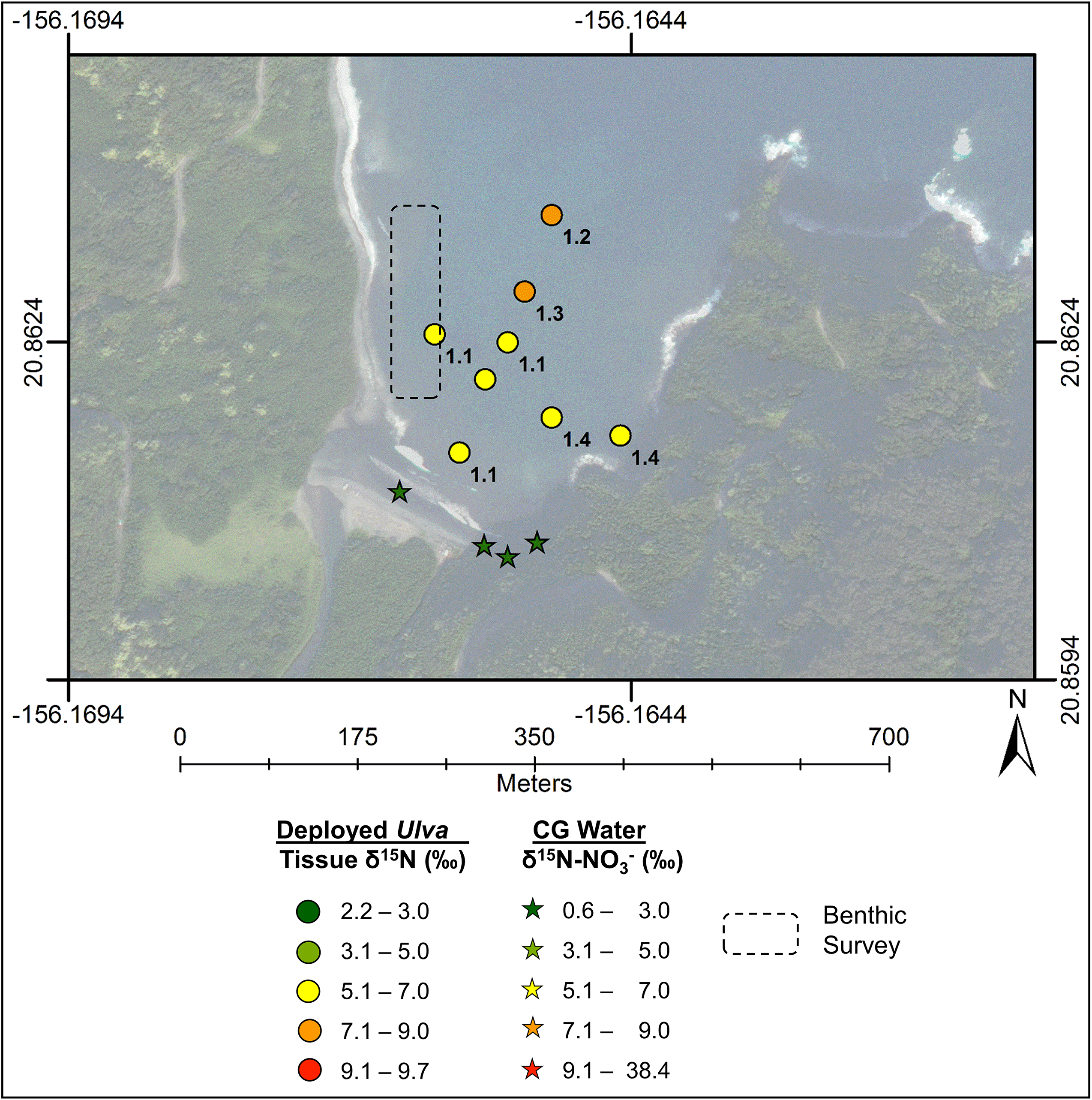

Supplement: S4 Fig — Sites of Ulva deployments and coastal groundwater samples (CG Water) are shown as filled circles and stars, respectively. Symbol colors at these sites indicate sample δ15N (‰) values as shown above. Boldface numbers indicate tissue N % values for deployed and shore-collected samples. Satellite imagery was used with permission from Esri (DigitalGlobe, GeoEye, i-cubed, Earthstar Geographics, CNES/Airbus DS, USDA, USGS, AEX, Getmapping, Aerogrid, IGN, IGP, swisstopo, and Technology and the GIS User Community; All rights reserved). (TIF) [file pone.0165825.s005.tif]

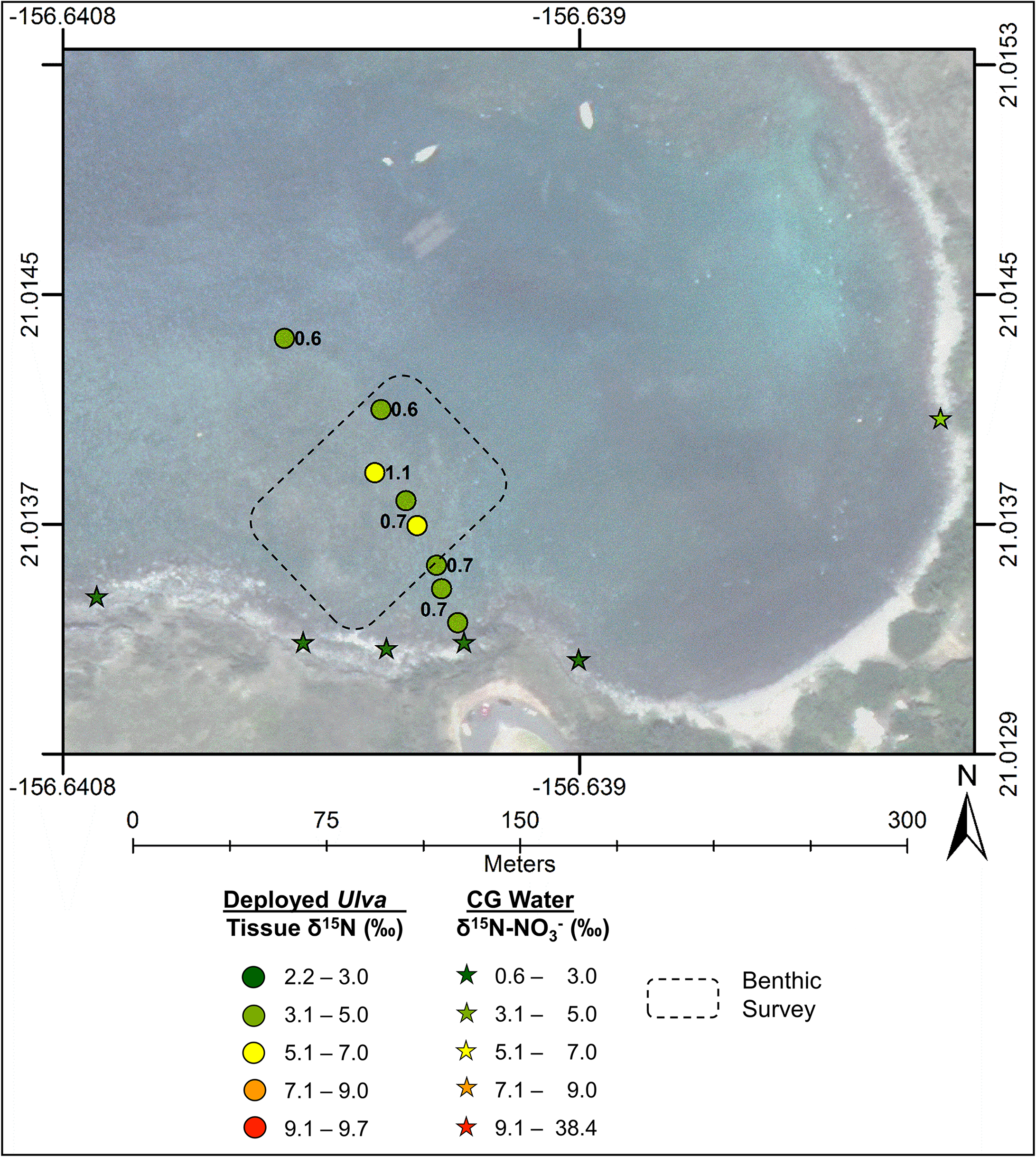

Supplement: S5 Fig — Sites of Ulva deployments and coastal groundwater samples (CG Water) are shown as filled circles and stars, respectively. Symbol colors at these sites indicate sample δ15N (‰) values as shown above. Satellite imagery was used with permission from Esri (DigitalGlobe, GeoEye, i-cubed, Earthstar Geographics, CNES/Airbus DS, USDA, USGS, AEX, Getmapping, Aerogrid, IGN, IGP, swisstopo, The University of Hawaiʻi School of Ocean and Earth Science and Technology and the GIS User Community; All rights reserved). (TIF) [file pone.0165825.s006.tif]

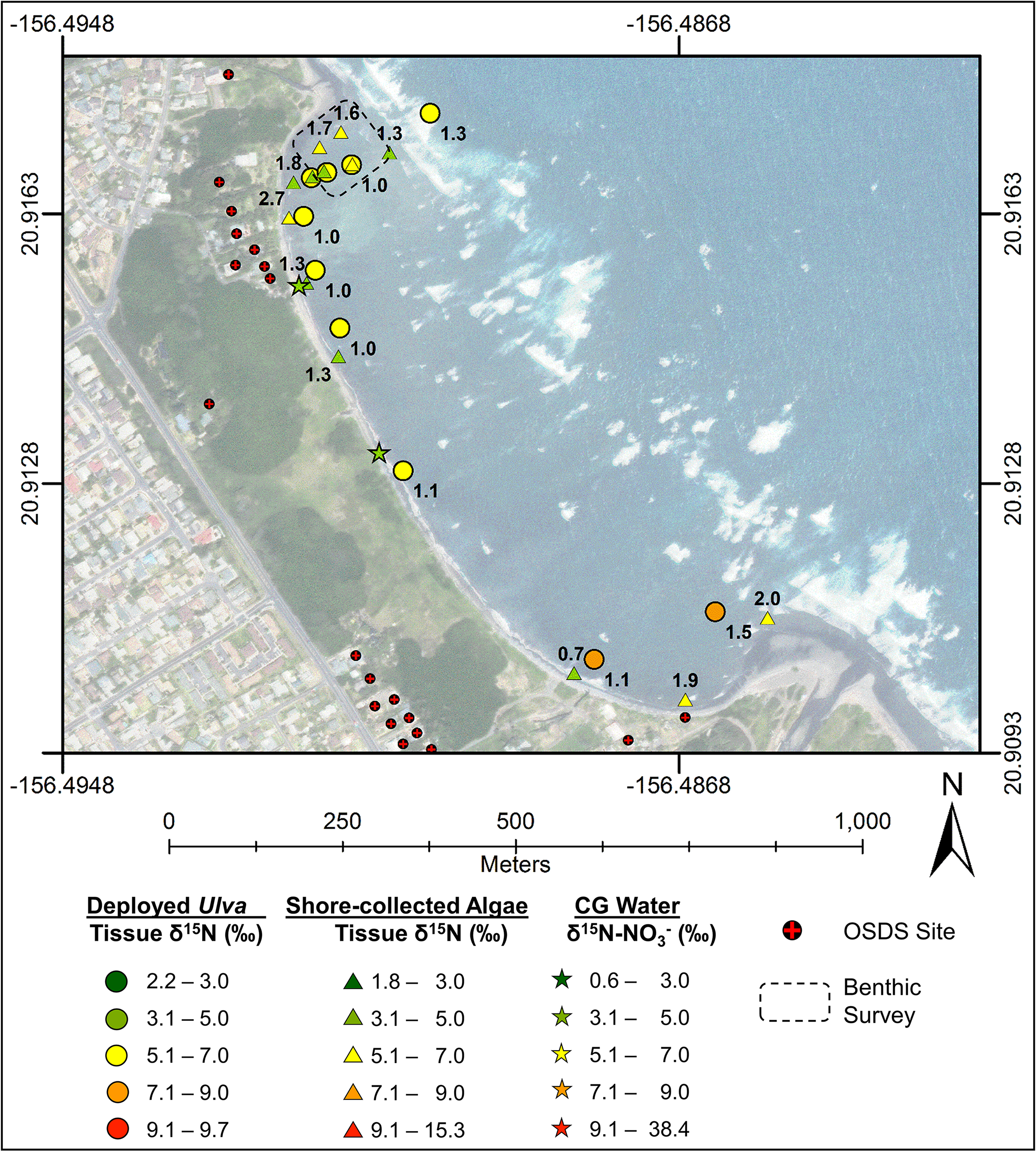

Supplement: S6 Fig — Sites of Ulva deployments, algal shore-collections, and coastal groundwater samples (CG Water) are shown as filled circles, triangles, and stars, respectively. Symbol colors at these sites indicate sample δ15N (‰) values as shown above. Boldface numbers indicate tissue N % values for deployed and shore-collected samples. Satellite imagery was used with permission (Esri, DigitalGlobe, GeoEye, i-cubed, Earthstar Geographics, CNES/Airbus DS, USDA, USGS, AEX, Getmapping, Aerogrid, IGN, IGP, swisstopo, The University of Hawaiʻi School of Ocean and Earth Science and Technology and the GIS User Community; All rights reserved). (TIF) [file pone.0165825.s007.tif]
