# Supplementary material for: Impact of Submarine Groundwater Discharge on Marine Water Quality and Reef Biota of Maui
Source: PLoS One. 2016 Nov 3;11(11):e0165825. doi: 10.1371/journal.pone.0165825 (PMC5094668; doi:10.1371/journal.pone.0165825)
Supplement: S1 Table — The correlation coefficient (rs) and p-value (p) are shown for correlations between salinity, silicate (SiO44-), total dissolved nitrogen (TDN), dissolved inorganic N (DIN), total dissolved phosphorous (TDP), and dissolved phosphate (PO43-). Marine surface water samples from study locations were pooled; n = 74. (DOCX) [file pone.0165825.s008.docx]

**S1 Table. Spearman’s correlation results for marine surface water samples.**

The correlation coefficient (r_s_) and p-value (p) is shown for parameters salinity, silicate (SiO_4_^4-^), total dissolved nitrogen (TDN), dissolved inorganic N (DIN), total dissolved phosphorous (TDP), and dissolved phosphate (PO_4_^3-^). Marine surface water samples from study locations were pooled; n = 74.

|  |  | **SiO_4_^4-^** | **TDN** | **DIN** | **TDP** | **PO_4_^3-^** |
| --- | --- | --- | --- | --- | --- | --- |
| **Salinity** | r_s_ | -0.50 | -0.38 | -0.40 | -0.42 | -0.49 |
|  | p | 0.00000632 | 0.000955 | 0.000481 | 0.000185 | 0.0000136 |
|  |  |  |  |  |  |  |
| **SiO_4_^4-^** | r_s_ |  | 0.38 | 0.67 | 0.56 | 0.93 |
|  | p |  | 0.000995 | 0.0000002 | 0.000000353 | 0.0000002 |
|  |  |  |  |  |  |  |
| **TDN** | r_s_ |  |  | 0.68 | 0.53 | 0.32 |
|  | p |  |  | 0.0000002 | 0.0000016 | 0.00523 |
|  |  |  |  |  |  |  |
| **DIN** | r_s_ |  |  |  | 0.56 | 0.65 |
|  | p |  |  |  | 0.000000344 | 0.0000002 |
|  |  |  |  |  |  |  |
| **TDP** | r_s_ |  |  |  |  | 0.64 |
|  | p |  |  |  |  | 0.0000002 |
|  |  |  |  |  |  |  |
