# Supplementary material for: Impact of Submarine Groundwater Discharge on Marine Water Quality and Reef Biota of Maui
Source: PLoS One. 2016 Nov 3;11(11):e0165825. doi: 10.1371/journal.pone.0165825 (PMC5094668; doi:10.1371/journal.pone.0165825)
Supplement: S2 Table — Water samples were collected adjacent to deployment cages at Honolua Bay. The correlation coefficient (rs) and p-value (p) are shown for correlations between distance from shore (distance) in meters, salinity, silicate (SiO44-), total dissolved nitrogen (TDN), dissolved inorganic N (DIN), total dissolved phosphorous (TDP), and dissolved phosphate (PO43-). n = 9. (DOCX) [file pone.0165825.s009.docx]

|  |  | **Salinity** | **SiO_4_^4-^** | **TDN** | **DIN** | **TDP** | **PO_4_^3-^** |
| --- | --- | --- | --- | --- | --- | --- | --- |
| **Distance** | r_s_ | 0.48 | -0.95 | 0.13 | -0.90 | -0.32 | -0.85 |
|  | p | 0.169 | 0.0000002 | 0.709 | 0.0000002 | 0.381 | 0.000392 |
|  |  |  |  |  |  |  |  |
| **Salinity** | r_s_ |  | -0.56 | -0.12 | -0.44 | -0.22 | -0.28 |
|  | p |  | 0.0988 | 0.742 | 0.223 | 0.55 | 0.434 |
|  |  |  |  |  |  |  |  |
| **SiO_4_^4-^** | r_s_ |  |  | 0.12 | 0.95 | 0.49 | 0.90 |
|  | p |  |  | 0.742 | 0.0000002 | 0.169 | 0.0000002 |
|  |  |  |  |  |  |  |  |
| **TDN** | r_s_ |  |  |  | 0.18 | 0.66 | 0.18 |
|  | p |  |  |  | 0.612 | 0.0428 | 0.612 |
|  |  |  |  |  |  |  |  |
| **DIN** | r_s_ |  |  |  |  | 0.58 | 0.98 |
|  | p |  |  |  |  | 0.0874 | 0.0000002 |
|  |  |  |  |  |  |  |  |
| **TDP** | r_s_ |  |  |  |  |  | 0.638 |
|  | p |  |  |  |  |  | 0.0583 |

**S2 Table. Spearman’s correlation results for marine surface water at Honolua Bay.**

Samples were collected adjacent to deployment cages at Honolua Bay. The correlation coefficient (r_s_) and p-value (p) is shown for parameters distance from shore (distance) in meters, salinity, silicate (SiO_4_^4-^), total dissolved nitrogen (TDN), dissolved inorganic N (DIN), total dissolved phosphorous (TDP), and dissolved phosphate (PO_4_^3-^). n = 9.
