# Supplementary material for: Impact of Submarine Groundwater Discharge on Marine Water Quality and Reef Biota of Maui
Source: PLoS One. 2016 Nov 3;11(11):e0165825. doi: 10.1371/journal.pone.0165825 (PMC5094668; doi:10.1371/journal.pone.0165825)
Supplement: S3 Table — Water samples were collected adjacent to deployment cages at Honomanū Bay. The correlation coefficient (rs) and p-value (p) are shown for correlations between distance from shore (distance) in meters, salinity, silicate (SiO44-), total dissolved nitrogen (TDN), dissolved inorganic N (DIN), total dissolved phosphorous (TDP), and dissolved phosphate (PO43-). n = 9. (DOCX) [file pone.0165825.s010.docx]

**S3 Table.** **Spearman’s correlation results for marine surface water at Honomanū Bay.**

|  |  | **Salinity** | **SiO_4_^4-^** | **TDN** | **DIN** | **TDP** | **PO_4_^3-^** |
| --- | --- | --- | --- | --- | --- | --- | --- |
| **Distance** | r_s_ | 0.45 | -0.53 | -0.60 | -0.60 | -0.82 | -0.73 |
|  | p | 0.204 | 0.124 | 0.0769 | 0.0769 | 0.00393 | 0.02 |
|  |  |  |  |  |  |  |  |
| **Salinity** | r_s_ |  | -0.80 | -0.77 | -0.89 | -0.80 | -0.58 |
|  | p |  | 0.00625 | 0.0121 | 0.0000002 | 0.00625 | 0.0874 |
|  |  |  |  |  |  |  |  |
| **SiO_4_^4-^** | r_s_ |  |  | 0.65 | 0.80 | 0.82 | 0.88 |
|  | p |  |  | 0.0501 | 0.00625 | 0.00393 | 0.0000002 |
|  |  |  |  |  |  |  |  |
| **TDN** | r_s_ |  |  |  | 0.82 | 0.73 | 0.63 |
|  | p |  |  |  | 0.00393 | 0.02 | 0.0583 |
|  |  |  |  |  |  |  |  |
| **DIN** | r_s_ |  |  |  |  | 0.76 | 0.65 |
|  | p |  |  |  |  | 0.0121 | 0.0501 |
|  |  |  |  |  |  |  |  |
| **TDP** | r_s_ |  |  |  |  |  | 0.817 |
|  | p |  |  |  |  |  | 0.00393 |

Samples were collected adjacent to deployment cages at Honomanū Bay. The correlation coefficient (r_s_) and p-value (p) is shown for parameters distance from shore (distance) in meters, salinity, silicate (SiO_4_^4-^), total dissolved nitrogen (TDN), dissolved inorganic N (DIN), total dissolved phosphorous (TDP), and dissolved phosphate (PO_4_^3-^). n = 9
