# Supplementary material for: Impact of Submarine Groundwater Discharge on Marine Water Quality and Reef Biota of Maui
Source: PLoS One. 2016 Nov 3;11(11):e0165825. doi: 10.1371/journal.pone.0165825 (PMC5094668; doi:10.1371/journal.pone.0165825)
Supplement: S4 Table — Water samples were collected adjacent to deployment cages at Kahului Bay. The correlation coefficient (rs) and p-value (p) are shown for correlations between distance from Kahului WRF (distance) in meters, salinity, silicate (SiO44-), total dissolved nitrogen (TDN), dissolved inorganic N (DIN), total dissolved phosphorous (TDP), and dissolved phosphate (PO43-). n = 16. (DOCX) [file pone.0165825.s011.docx]

**S4 Table. Spearman’s correlation results for marine surface water at Kahului Bay.**

|  |  | **Salinity** | **SiO_4_^4-^** | **TDN** | **DIN** | **TDP** | **PO_4_^3-^** |
| --- | --- | --- | --- | --- | --- | --- | --- |
| **Distance** | r_s_ | -0.02 | -0.11 | -0.02 | 0.24 | 0.34 | -0.06 |
|  | p | 0.935 | 0.68 | 0.935 | 0.366 | 0.198 | 0.814 |
|  |  |  |  |  |  |  |  |
| **Salinity** | r_s_ |  | -0.335 | -0.515 | -0.374 | -0.361 | -0.188 |
|  | p |  | 0.198 | 0.0402 | 0.149 | 0.163 | 0.476 |
|  |  |  |  |  |  |  |  |
| **SiO_4_^4-^** | r_s_ |  |  | 0.0941 | 0.741 | 0.0981 | 0.965 |
|  | p |  |  | 0.72 | 0.00 | 0.71 | 0.00 |
|  |  |  |  |  |  |  |  |
| **TDN** | r_s_ |  |  |  | 0.20 | 0.44 | 0.02 |
|  | p |  |  |  | 0.456 | 0.0866 | 0.935 |
|  |  |  |  |  |  |  |  |
| **DIN** | r_s_ |  |  |  |  | 0.364 | 0.774 |
|  | p |  |  |  |  | 0.16 | 0.0000328 |
|  |  |  |  |  |  |  |  |
| **TDP** | r_s_ |  |  |  |  |  | 0.142 |
|  | p |  |  |  |  |  | 0.594 |

Samples were collected adjacent to deployment cages at Kahului Bay. The correlation coefficient (r_s_) and p-value (p) is shown for parameters distance from Kahului WWRF (distance) in meters, salinity, silicate (SiO_4_^4-^), total dissolved nitrogen (TDN), dissolved inorganic N (DIN), total dissolved phosphorous (TDP), and dissolved phosphate (PO_4_^3-^). n = 16
