# Supplementary material for: Impact of Submarine Groundwater Discharge on Marine Water Quality and Reef Biota of Maui
Source: PLoS One. 2016 Nov 3;11(11):e0165825. doi: 10.1371/journal.pone.0165825 (PMC5094668; doi:10.1371/journal.pone.0165825)
Supplement: S5 Table — Water samples were collected adjacent to deployment cages at Māʻalaea Bay. The correlation coefficient (rs) and p-value (p) are shown for correlations between distance from shore (distance) in meters, salinity, silicate (SiO44-), total dissolved nitrogen (TDN), dissolved inorganic N (DIN), total dissolved phosphorous (TDP), and dissolved phosphate (PO43-). n = 10. (DOCX) [file pone.0165825.s012.docx]

**S5 Table. Spearman’s correlation results for marine surface water at Māʻalaea Bay.**

|  |  | **Salinity** | **SiO_4_^4-^** | **TDN** | **DIN** | **TDP** | **PO_4_^3-^** |
| --- | --- | --- | --- | --- | --- | --- | --- |
| **Distance** | r_s_ | 0.81 | -0.50 | -0.78 | -0.67 | -0.63 | -0.69 |
|  | p | 0.00257 | 0.126 | 0.00523 | 0.029 | 0.048 | 0.0252 |
|  |  |  |  |  |  |  |  |
| **Salinity** | r_s_ |  | -0.61 | -0.75 | -0.73 | -0.69 | -0.66 |
|  | p |  | 0.0537 | 0.0108 | 0.0131 | 0.0252 | 0.0332 |
|  |  |  |  |  |  |  |  |
| **SiO_4_^4-^** | r_s_ |  |  | 0.75 | 0.88 | 0.84 | 0.87 |
|  | p |  |  | 0.0108 | 0.0000002 | 0.0000002 | 0.0000002 |
|  |  |  |  |  |  |  |  |
| **TDN** | r_s_ |  |  |  | 0.81 | 0.69 | 0.84 |
|  | p |  |  |  | 0.00257 | 0.0252 | 0.0000002 |
|  |  |  |  |  |  |  |  |
| **DIN** | r_s_ |  |  |  |  | 0.97 | 0.94 |
|  | p |  |  |  |  | 0.0000002 | 0.0000002 |
|  |  |  |  |  |  |  |  |
| **TDP** | r_s_ |  |  |  |  |  | 0.919 |
|  | p |  |  |  |  |  | 0.0000002 |

Samples were collected adjacent to deployment cages at Māʻalaea Bay. The correlation coefficient (r_s_) and p-value (p) is shown for parameters distance from shore (distance) in meters, salinity, silicate (SiO_4_^4-^), total dissolved nitrogen (TDN), dissolved inorganic N (DIN), total dissolved phosphorous (TDP), and dissolved phosphate (PO_4_^3-^). n = 10
