# Supplementary material for: Impact of Submarine Groundwater Discharge on Marine Water Quality and Reef Biota of Maui
Source: PLoS One. 2016 Nov 3;11(11):e0165825. doi: 10.1371/journal.pone.0165825 (PMC5094668; doi:10.1371/journal.pone.0165825)
Supplement: S7 Table — Water samples were collected adjacent to deployment cages at Waiehu Bay. The correlation coefficient (rs) and p-value (p) are shown for correlations between distance from shore (distance) in meters, salinity, silicate (SiO44-), total dissolved nitrogen (TDN), dissolved inorganic N (DIN), total dissolved phosphorous (TDP), and dissolved phosphate (PO43-). n = 10. (DOCX) [file pone.0165825.s014.docx]

|  |  | **Salinity** | **SiO_4_^4-^** | **TDN** | **DIN** | **TDP** | **PO_4_^3-^** |
| --- | --- | --- | --- | --- | --- | --- | --- |
| **Distance** | r_s_ | 0.71 | -0.75 | -0.82 | -0.19 | -0.59 | -0.56 |
|  | p | 0.0186 | 0.0108 | 0.0015 | 0.583 | 0.0665 | 0.0812 |
|  |  |  |  |  |  |  |  |
| **Salinity** | r_s_ |  | -0.99 | -0.53 | -0.12 | -0.39 | -0.84 |
|  | p |  | 0.0000002 | 0.107 | 0.733 | 0.243 | 0.0000002 |
|  |  |  |  |  |  |  |  |
| **SiO_4_^4-^** | r_s_ |  |  | 0.59 | 0.07 | 0.39 | 0.86 |
|  | p |  |  | 0.0665 | 0.838 | 0.243 | 0.0000002 |
|  |  |  |  |  |  |  |  |
| **TDN** | r_s_ |  |  |  | 0.30 | 0.32 | 0.41 |
|  | p |  |  |  | 0.384 | 0.346 | 0.213 |
|  |  |  |  |  |  |  |  |
| **DIN** | r_s_ |  |  |  |  | 0.34 | -0.10 |
|  | p |  |  |  |  | 0.327 | 0.759 |
|  |  |  |  |  |  |  |  |
| **TDP** | r_s_ |  |  |  |  |  | 0.107 |
|  | p |  |  |  |  |  | 0.759 |

**S7 Table.** **Spearman’s correlation results for marine surface water at Waiehu Bay.**

Samples were collected adjacent to deployment cages at Waiehu Bay. The correlation coefficient (r_s_) and p-value (p) is shown for parameters distance from shore (distance) in meters, salinity, silicate (SiO_4_^4-^), total dissolved nitrogen (TDN), dissolved inorganic N (DIN), total dissolved phosphorous (TDP), and dissolved phosphate (PO_4_^3-^). n = 10
